# Web-Based Cognitive Behavioral Therapy for Female Patients With Eating Disorders: Randomized Controlled Trial

**DOI:** 10.2196/jmir.3946

**Published:** 2015-06-18

**Authors:** Elke D ter Huurne, Hein A de Haan, Marloes G Postel, Job van der Palen, Joanne EL VanDerNagel, Cornelis AJ DeJong

**Affiliations:** ^1^ Tactus Addiction Treatment Enschede Netherlands; ^2^ Nijmegen Institute for Scientist-Practitioners in Addiction Nijmegen Netherlands; ^3^ University of Twente Department of Psychology, Health & Technology Enschede Netherlands; ^4^ Medisch Spectrum Twente Medical School Twente Enschede Netherlands; ^5^ University of Twente Department of Research Methodology, Measurement and Data Analysis Enschede Netherlands; ^6^ Aveleijn Borne Netherlands; ^7^ Radboud University Behavioral Science Institute Nijmegen Netherlands

**Keywords:** eating disorders, bulimia nervosa, binge-eating disorder, eating disorders not otherwise specified, randomized controlled trial, eHealth, Web-based treatment, asynchronous therapeutic support, treatment effectiveness, cognitive behavioral therapy

## Abstract

**Background:**

Many patients with eating disorders do not receive help for their symptoms, even though these disorders have severe morbidity. The Internet may offer alternative low-threshold treatment interventions.

**Objective:**

This study evaluated the effects of a Web-based cognitive behavioral therapy (CBT) intervention using intensive asynchronous therapeutic support to improve eating disorder psychopathology, and to reduce body dissatisfaction and related health problems among patients with eating disorders.

**Methods:**

A two-arm open randomized controlled trial comparing a Web-based CBT intervention to a waiting list control condition (WL) was carried out among female patients with bulimia nervosa (BN), binge eating disorder (BED), and eating disorders not otherwise specified (EDNOS). The eating disorder diagnosis was in accordance with the *Diagnostic and Statistical Manual of Mental Disorders*, 4th edition, and was established based on participants’ self-report. Participants were recruited from an open-access website, and the intervention consisted of a structured two-part program within a secure Web-based application. The aim of the first part was to analyze participant’s eating attitudes and behaviors, while the second part focused on behavioral change. Participants had asynchronous contact with a personal therapist twice a week, solely via the Internet. Self-report measures of eating disorder psychopathology (primary outcome), body dissatisfaction, physical health, mental health, self-esteem, quality of life, and social functioning were completed at baseline and posttest.

**Results:**

A total of 214 participants were randomized to either the Web-based CBT group (n=108) or to the WL group (n=106) stratified by type of eating disorder (BN: n=44; BED: n=85; EDNOS: n=85). Study attrition was low with 94% of the participants completing the posttest assignment. Overall, Web-based CBT showed a significant improvement over time for eating disorder psychopathology (*F*_97_=63.07, *P*<.001, *d*=.82) and all secondary outcome measures (effect sizes between *d*=.34 to *d*=.49), except for Body Mass Index. WL participants also improved on most outcomes; however, effects were smaller in this group with significant between-group effects for eating disorder psychopathology (*F*_201_=9.42, *P*=.002, *d*=.44), body dissatisfaction (*F*_201_=13.16, *P*<.001, *d*=.42), physical health (*F*_200_=12.55, *P*<.001, *d*=.28), mental health (*F*_203_=4.88, *P*=.028, *d*=.24), self-esteem (*F*_202_=5.06, *P*=.026, *d*=.20), and social functioning (*F*_205_=7.93, *P*=.005, *d*=.29). Analyses for the individual subgroups BN, BED, and EDNOS showed that eating disorder psychopathology improved significantly over time among Web-based CBT participants in all three subgroups; however, the between-group effect was significant only for participants with BED (*F*_78_=4.25, *P*=.043, *d*=.61).

**Conclusions:**

Web-based CBT proved to be effective in improving eating disorder psychopathology and related health among female patients with eating disorders.

**Trial Registration:**

Nederlands Trial Register (NTR): NTR2415; http://www.trialregister.nl/trialreg/admin/rctview.asp?TC=2415 (Archived by WebCite at http://www.webcitation.org/6T2io3DnJ).

## Introduction

In the Netherlands, eating disorders have a lifetime prevalence of 1.74% [1], and these disorders account for severe psychological, physical, and social morbidity. Although early identification and treatment is desired, patients often refrain from seeking or receiving help because of personal barriers, such as feelings of shame and fear of stigmatization, and intervention-related barriers, such as costs, geographical distance, and lack of availability [2-6]. Psychiatric services are challenged to help patients overcome these barriers by providing easily accessible, low-threshold interventions.

The Internet offers many possibilities for these types of interventions because of its relative anonymity, widespread and 24-hour access, and increasing usage. Low-threshold Internet interventions may reach patients with less advanced disorders and prevent their condition from progressing. Moreover, Web-based interventions for psychopathology such as depression, anxiety, and addictive behaviors have already proven successful, with interventions using (intensive) therapeutic support being more effective than self-help or minimal contact interventions [7-9]. In the past few years, several Internet interventions have been developed for patients with eating disorders, and a recent review showed that these treatments can be effective in reducing eating disorder psychopathology, binge eating, and purging, as well as in improving quality of life [10]. However, it should be noted that most studies were conducted among patients with bulimia nervosa (BN) and (to a lesser extent) binge eating disorder (BED) [10], whereas eating disorders not otherwise specified (EDNOS) is the most commonly diagnosed eating disorder [11]. Additionally, studies conducted among patients with EDNOS mostly included interventions aimed at (indicated) prevention or early intervention in eating disorders [12-14]. Although some interventions proved to be effective, most studies included only adolescents and young women [12,13], while the EDNOS subgroup includes a broader population of patients with eating disorder symptoms. Therefore, in 2009 a Web-based cognitive behavioral therapy (CBT) intervention with intensive therapeutic support was developed to treat Dutch patients with all types of eating disorders [15], based on a similar intervention for problem drinkers [16,17], in which patients communicate asynchronously with their therapist twice a week. A before-after study into this intervention showed a reduction in eating disorder psychopathology (*d*=1.14) and body dissatisfaction (*d*=0.86), as well as high patient satisfaction [18]. However, this study had a nonrandomized design and included only those participants who completed the intervention (54% of participants).

This study, therefore, aimed to explore the effects of the Web-based CBT intervention, compared to a waiting list control group (WL), on eating disorder psychopathology (primary outcome) as well as body dissatisfaction, physical health, body mass index (BMI), mental health, self-esteem, quality of life, and social functioning (secondary outcomes). Furthermore, we were interested in the effects of the Web-based CBT across participants of the specific eating disorder subgroups: BN, BED, and EDNOS.

## Methods

### Participants

**Figure 1:**
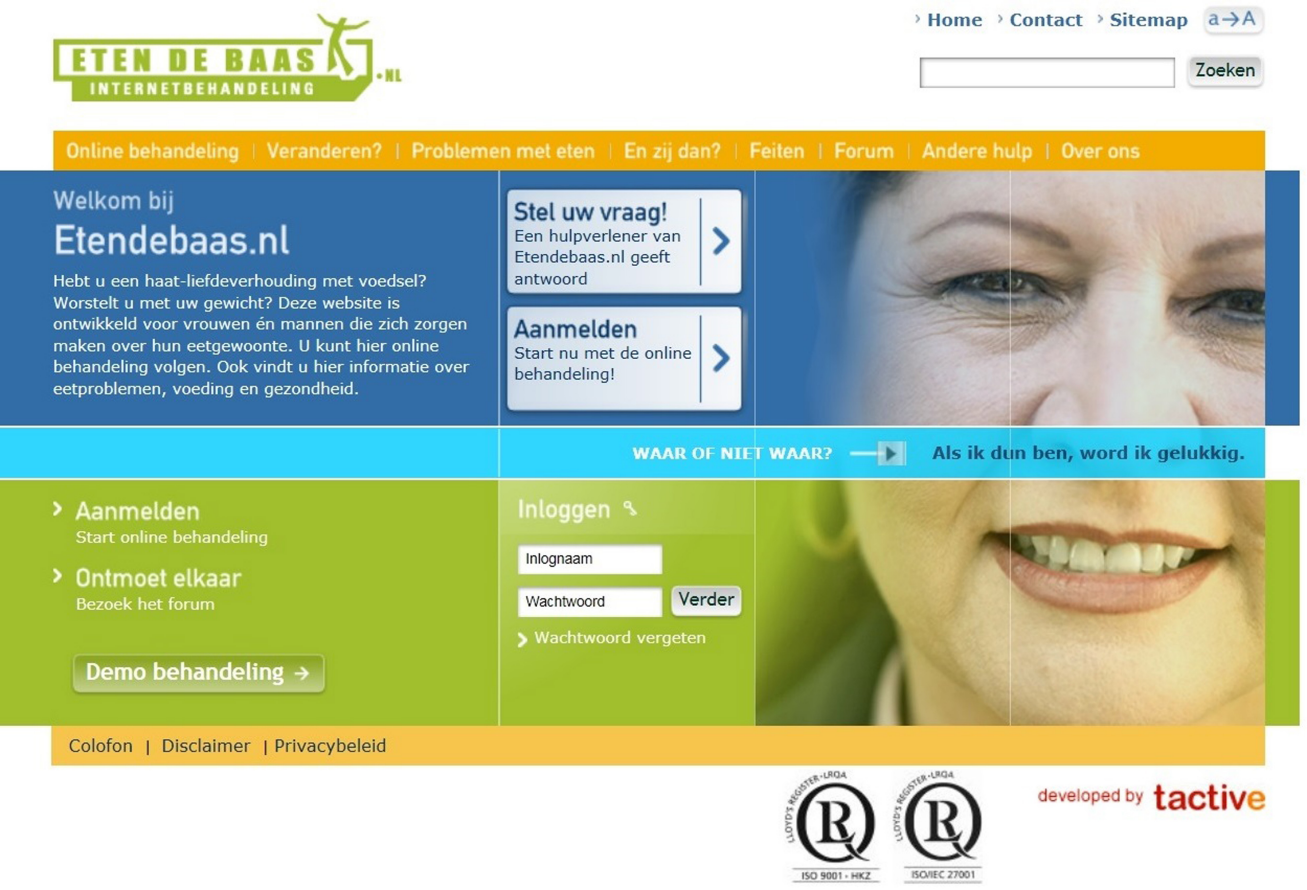
Website homepage.

Participants were self-recruited users of the Dutch website “Look at your eating” [19]. This open-access website (see Figure 1) offered general information on eating disorders and related topics, a forum for peer support, as well as information about the Web-based CBT program and the study procedures of this trial. Inclusion criteria for participation were (1) female gender, (2) age ≥18 years, (3) diagnosis of BN, BED, or EDNOS (based on participants’ self-report), (4) written and oral fluency in Dutch language, (5) access to Internet, (6) signed informed consent, and (7) a referral from a general practitioner (GP). Exclusion criteria were (1) severe underweight, (2) suicidal ideation, (3) receiving psychological or pharmaceutical treatment for any eating disorder within the past 6 months, (4) pregnancy, and (5) expected absence for 4 weeks or longer during the intervention period (eg, due to planned vacation). If participation in the intervention was not possible for some reason (eg, lack of Dutch health insurance and therefore funding of the intervention, or patient’s GP did not agree with participation), patients were also not eligible for this study.

### Study Design and Procedure

**Figure 2:**
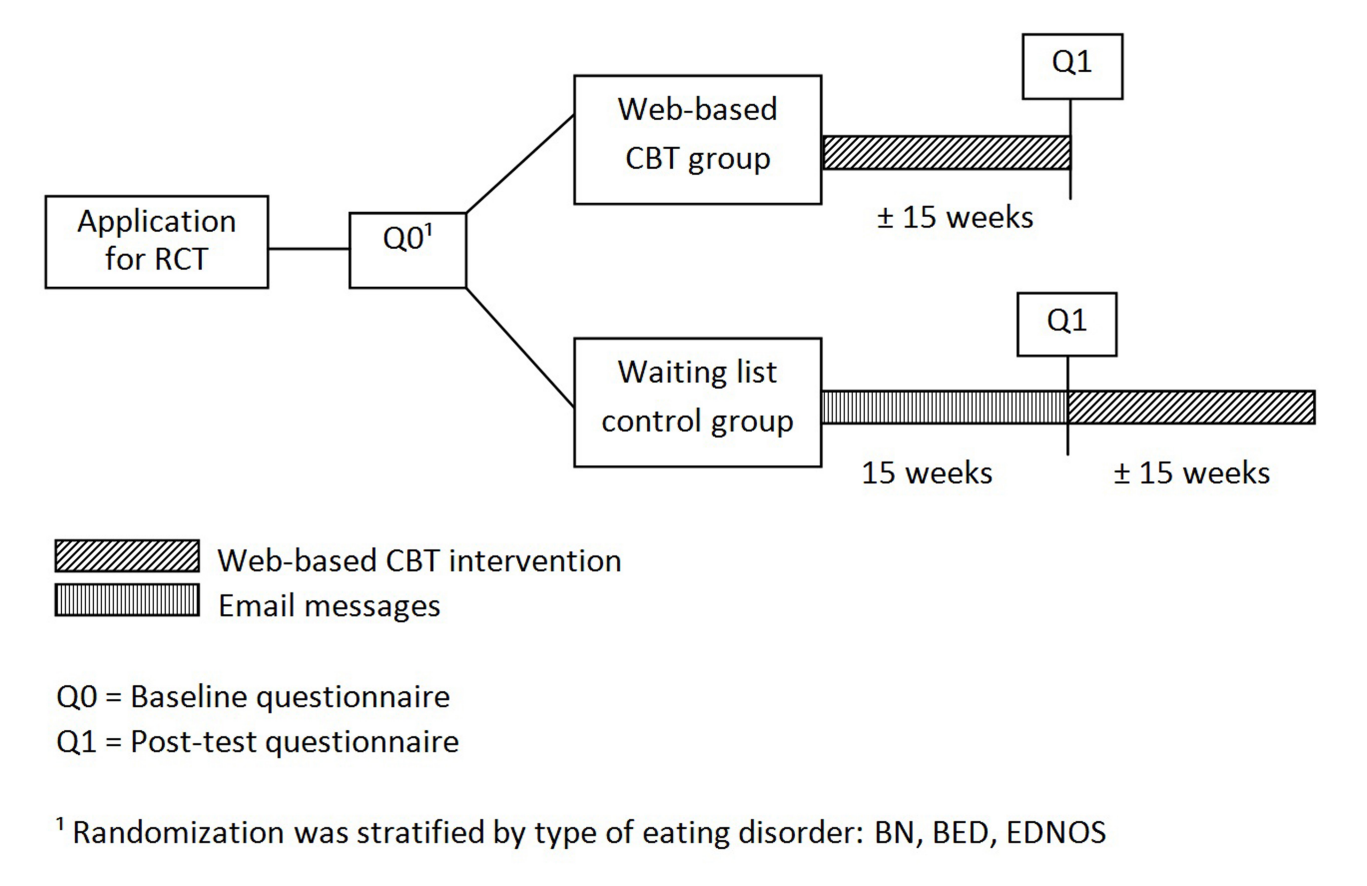
Flow chart of study design and timeline.

This study was a randomized controlled trial with two groups: Web-based cognitive behavioral therapy (Web-based CBT) and waiting list control (WL). Figure 2 presents a flow chart of the design and timeline of the study. Recruitment took place from March 2011 until December 2013. Information about the study was disseminated through announcements on eating disorder–related websites and forums, and newspaper advertisements. Website visitors were invited to read the information about the study explicitly, provide their email addresses and telephone numbers, and agree with the conditions of the Web-based CBT protocol. Furthermore, they had to provide written informed consent, personal data, and data of their GP. The GP was informed about the patients’ participation in the study (as covered by the Ethics Committee approval) and requested to sign and return the referral form. To assess eligibility and to obtain baseline data, participants completed an online self-report questionnaire during the sign-up procedure. Based on this questionnaire, the *Diagnostic and Statistical Manual of Mental Disorders,* 4th Edition (DSM-IV) eating disorder diagnosis was assessed and randomization took place. Participants not eligible for this study were offered participation in the regular Web-based CBT intervention (outside this study). This was possible only by logging in with a personal code that individuals received by mail after providing personal data. In case of urgent medical risks, no funding of the intervention, or disagreement of the GP for participation in the intervention, participants were referred to their GP or advised on more appropriate treatment.

Participants were randomized to the Web-based CBT or WL through computer-generated randomly varying block sizes (2, 4, or 8), stratified by type of eating disorder (BN, BED, EDNOS). Randomization was performed at a 1:1 ratio. The allocation schedule was prepared by an independent researcher not involved in data collection. The assignment of participants to the conditions was not dependent on the participants’ characteristics. Participants assigned to the Web-based CBT started the intervention immediately while participants of the WL had to wait 15 weeks, during which they received supportive email messages once every 2 weeks to keep them involved. All WL participants were guaranteed treatment after the waiting period, and they were advised to contact their GP in case earlier treatment was needed.

To measure efficacy, the WL group completed the posttest questionnaire after the 15-week waiting period, and the Web-based CBT group completed this questionnaire after completing or prematurely ending the intervention, or in case of a longer treatment duration, 18 weeks after randomization (to keep the time frame between the first and second assessments as close as possible in both groups). The posttest questionnaire measured all primary and secondary outcomes and included evaluation questions about the Web-based CBT intervention and treatment non-completion when applicable. Participants received a €10 digital voucher for an online store for each completed questionnaire, except for the baseline questionnaire.

The study protocol was approved by the Ethics Committee of Medical Spectrum Twente in March 2011 (reference number NL31717.044.010, P10-31) and was registered on the Dutch Trial Registry (NTR2415). Details of this protocol have been published previously [20]. See Multimedia Appendix 1 for the CONSORT-EHEALTH checklist [21].

### Web-Based Cognitive Behavioral Therapy Intervention

The Web-based CBT intervention [15,18] “Look at your eating” (in Dutch: Etendebaas) was developed at Tactus Addiction Treatment by health care professionals (psychologist, addiction medicine physician, psychotherapist, psychiatrist, dietician, registered nurses, and social workers), a software development team (The Factor-E), and patients and members of a Dutch organization for people with eating disorders. Development of the intervention was an interactive and iterative process, involving patients providing input and feedback on different versions of the content, layout, visual features, and ease of navigation of the Web-based CBT intervention. The intervention included a structured two-part program with at least 21 contact moments and 10 assignments within a secure Web-based application. Multimedia Appendix 2 presents an overview of the content of the intervention. The first part aimed to analyze participants’ eating attitudes and behaviors, while the second part focused on behavioral change. The modules were organized in a pre-specified order, and participants had access only to the information and assignments that were sent by the therapist at a specific point. CBT [22-24] and motivational interviewing (MI) [25,26] were the fundamentals of the intervention including techniques such as psycho-education, self-monitoring, thought restructuring, problem solving, and relapse prevention.

**Figure 3:**
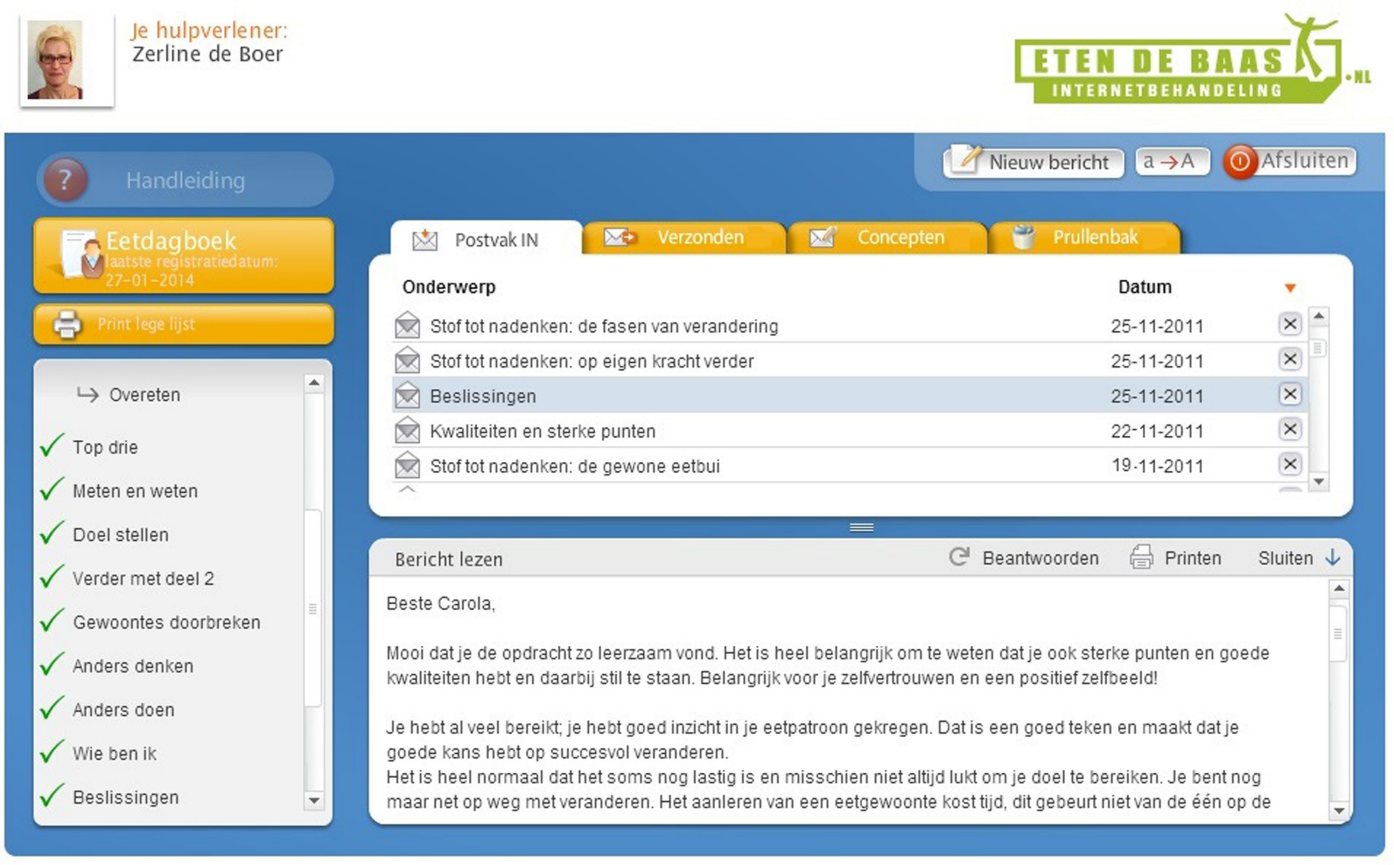
Participant's personal online file.

Participants had to log in to the application via the website [19] with a personal username and password, secured by Secure Sockets Layer, to have access to their personal file (see Figure 3). All data transferred between the participant’s computer and the application was encrypted and sent via the Hypertext Transfer Protocol Secure (https) protocol. The application was entirely server-based, and all information gathered was stored on an encrypted database. Daily backups of the server on an offsite location were made to ensure further data security.

During the Web-based CBT, participants normally had asynchronous contact twice a week with their personal therapists, solely via the Internet, unless participants specifically requested an additional telephone contact. Participation in the intervention took approximately 20 minutes per day, and participants were instructed about completing home assignments and registering eating behaviors daily in their online eating diary. Participants could access the intervention in their personal environment at any time, and in their private file they could read the messages from their therapist or complete assignments. Accordingly, therapists responded within 3 working days on the participants’ messages or assignments. The therapists’ messages were personalized to the participants’ situation but also comprised pre-programmed text fragments, for example, explaining the assignments. For each module, a format was available including the topics that should be addressed and the information that should be given by the therapist. Therapists could also include hyperlinks to information on the website [19] in their messages. These formats ensured consistency in the therapists’ messages. However, as all messages were adjusted to the situation of participants, with differences in complexity of personal issues, their content and length varied. Therapists always responded on participants’ completed eating diary, assignments, and questionnaires, but the content of these texts was dependent on what participants had filled out. The responses of the therapists were supportive and included also CBT and MI techniques. Moreover, their communication primarily focused on providing accurate and objective information, hopeful writing, reinforcement, and relabeling of demotivating statements. The progress of the participants was monitored by the therapists. When participants did not respond to the messages of the therapists within the next week, they received a reminder with a request to keep in touch regularly. When participants did not respond for 4 weeks, the intervention was terminated by the therapist. Participants were considered treatment completers if they had completed all 10 assignments and attended at least 21 sessions. In case participants stopped the intervention prematurely and still needed help, the therapist discussed with the participant what kind of treatment would be more appropriate. Possible options were face-to-face treatment with a professional (therapist or dietician), day care, or hospitalization in a specialist eating disorder institution. If preferred by the participant, the therapist also initiated the first contact with the other professional or institution.

A total of 17 therapists with either a bachelor’s degree in nursing or social work or a master’s degree in psychology were involved in this study. All therapists completed 2 days of training including theoretical information and practice-oriented assignments focusing on the design and implementation of the Web-based application, on technical aspects of delivering this intervention, and on using different strategies to apply the CBT and MI techniques (eg, restructuring of non-helpful thoughts into helpful thoughts, enhancing self-efficacy, expression of empathy, and evoking cognitive dissonance). Additionally, they also received a 1-day training session about eating disorders and related issues, and about the treatment content and protocol. Subsequently, all therapists completed a full treatment program with a test patient, and 3 months of intensive supervision by experienced coaches. A comprehensive manual to the Web-based CBT intervention was available for all therapists, which included a detailed description of the different modules of the intervention. Also safety protocols were described in this manual, covering the criteria for admission to part 2, as well as guidelines about what to do in case of severe eating problems, relapse or suicidal ideations, and when to inform the participant’s GP. Safety protocols were also included in the formats of the different modules. Besides the use of formats for all modules and the intensive training of therapists, several other methods were used to ensure quality and consistency in the treatment of participants. For example, all messages of the therapists were checked retrospectively (and adjusted if necessary) by a multidisciplinary team consisting of a psychologist, a psychotherapist, an addiction medicine physician, a psychiatrist, a dietician, and two coaches at the end of part 1 of the intervention. Furthermore, all patient files were regularly checked by the coaches of the Web-based CBT intervention and these coaches were also present daily for consultation and advice. The multidisciplinary team was available remotely for consultation throughout the intervention and gave also expert advice to the therapists at the end of part 1 of the intervention of each participant. For all participants, the intervention was covered by Dutch health insurance, although the costs were set off against participants’ deductible. Therefore, several of them needed to pay a contribution of up to €350 for their participation. This is a standard procedure in the Dutch health care system.

### Waiting List Control Group

Participants in the WL received seven supportive email messages sent by the researcher during the waiting period. The first message included a brief explanation about the design of the Web-based CBT intervention as well as information about when participants could start the intervention, the aim of the supporting email messages, and what participants should do in case they needed urgent help. The second message provided information about the different topics discussed on the website [19], such as information about factors that could effect participants’ eating behavior and information about the physical effects of eating disorders. The third email contained information about the online forum, which was part of the website [19] and provided contact with fellow sufferers. The fourth email included information about the scientific research project of the Web-based CBT intervention and the results of the pilot study. The fifth message contained a summary of comments from former participants of the Web-based CBT intervention (verbatim and anonymous) about their experiences with the treatment. The sixth email focused on common misconceptions about food, losing weight, and eating disorders. This message also referred to relevant information on the website [19] and possible consequences of these misconceptions. The last email included a description of the basic principles of the intervention and therapeutic support, and a description of what was expected of the participants during the intervention. Finally, participants were thanked for their patience during the waiting period and were wished success with their participation in the intervention. All email messages also concluded with information about what participants had to do when they needed urgent help. Specifically, they were referred to their GP in case of urgent medical problems. This information was also presented in the prior email that participants received the moment they were allocated to the control group.

### Measures

The primary outcome measure was eating disorder psychopathology measured with the global mean scale of the Eating Disorder Examination-Questionnaire (EDE-Q) [27], a widely used self-report questionnaire to measure eating disorder severity. Items were scored on a 7-point Likert scale (range 0-6), with a higher score reflecting more psychopathology. Additionally, the scores on the EDE-Q subscales Restraint, Eating Concern, Shape Concern, and Weight Concern were calculated. Secondary outcome measures included the Body Attitude Test (BAT) [28,29] to assess body dissatisfaction, the Maudsley Addiction Profile-Health Symptom Scale (MAP-HSS) [30] and 15 eating disorder-specific physical complaints to measure physical health, the Depression Anxiety Stress Scale (DASS) [31] to measure mental health, the Rosenberg Self-Esteem Scale (RSES) [32] to examine self-esteem, the EuroQol visual analogue scale (EQ-5D VAS) [33] to assess quality of life, the Measurements in the Addictions for Triage and Evaluation - International Classification of Functioning, Disability and Health (MATE-ICN) [34] to examine social functioning, and Body Mass Index (BMI). Participants completed these measures at baseline and posttest (approximately 15 weeks after baseline). Additionally, demographic data, DSM-IV diagnosis, prior care for eating disorders and other psychological problems, duration of illness, and suicide risk were measured at baseline. DSM-IV eating disorder diagnosis was assessed using the online self-report questionnaire Eating Disorder Questionnaire-Online (EDQ-O) and related baseline data, optionally with additional questions by email. The EDQ-O was developed as a diagnostic instrument for establishing all DSM-IV-TR eating disorder diagnoses without using face-to-face contact, as in-person clinical interviews are not suitable for Web-based interventions. A recent study into the validity of the EDQ-O showed that this self-report questionnaire performs acceptably as a diagnostic instrument for all eating disorder classifications [35]. However, as the results of the validation study were not available at the start of the current RCT, the EDQ-O was not used as the only tool to set a DSM-IV eating disorder classification in this study, but also other baseline data of participants were taken into consideration such as their BMI and completed EDE-Q. If there were doubts about the diagnosis established with the EDQ-O based on the combination of all data, participants were asked additional questions by email. Based on the responses of the participants, a final diagnosis was established by the psychologist and researcher. Suicide risk was measured using Part C of the MINI-Plus [36,37], consisting of 6 self-report items examining suicidal tendencies. Participants for whom the current risk of suicide was classified as “high” were excluded from the study. The exclusion criterion of severe underweight was assessed in case the body weight of participants was less than 85% of ideal body weight, determined using the table of height/weight limits of the MINI-Plus [36,37]. At posttest, participants were also asked if they had other support for their eating disorder during the intervention or waiting period. Furthermore, participants’ evaluation of the intervention and their personal therapist was measured as well as reasons for non-completion (if applicable).

### Statistical Analyses

Our sample size was calculated based on an expected mean difference score of 1.0 (SD 1.2) on the EDE-Q global score (primary outcome measure) between the Web-based CBT and WL at posttest. This expected difference was based on the results of our before-after study, adjusted for an estimated improvement in the WL. Power analysis (G*Power) revealed a sample size of 25 participants in each condition based on a significance level of 5%, a power of 80%, the same number of participants per condition, 2 measurements, and a correlation among repeated measures of 0.95. However, we expected 40% of the participants not to complete the Web-based CBT, therefore, 42 participants in each condition (Web-based CBT and WL) were needed. To determine the efficacy of the Web-based CBT for the specific eating disorder subtypes, the total sample size was determined at 84 participants with BN, 84 participants with BED, and 84 participants with EDNOS (total of 252 participants).

All analyses were conducted using SPSS version 21. Data are presented as numbers (percentage) for categorical data and as the means (SD) for continuous data. Baseline differences between the Web-based CBT and WL are expressed as differences in proportion for categorical data and as the mean differences for continuous data. Chi square or Fisher’s exact tests (as appropriate) were used to compare categorical measures between the groups, and *t* tests or Mann-Whitney tests to compare continuous measures. To measure baseline differences between the three subgroups, Chi square or Fisher’s exact tests were used to compare categorical measures, and analysis of variance (ANOVA) with Tukey’s post-hoc tests or Kruskall-Wallis tests were used to compare continuous measures. Post-hoc tests for categorical variables were conducted by pairwise comparisons, with a Holm-Bonferroni post-hoc correction.

To measure the efficacy of the Web-based CBT in terms of primary and secondary outcome measures, Mixed Models for repeated measures were used, allowing for the inclusion of all participants, regardless of missing data. The intervention*time interaction effect was used to measure whether the change over time was different for the Web-based CBT compared to the WL. Between-group effect sizes were calculated according to Cohen’s *d* by subtracting the average difference score between pretest and posttest of the control group from the corresponding difference score of the Web-based CBT group, and dividing the result by the pooled standard deviation of the pretest. Additionally, the effects over time within the Web-based CBT and WL group were measured. Within-group effect sizes were calculated by subtracting the average score at posttest from the average score at pretest and dividing the result by the pooled standard deviation of the pretest. Effect sizes of 0.8 were considered large, effect sizes of 0.5 moderate, and effect sizes of 0.2 small [38].

## Results

### Participant Flow

**Figure 4:**
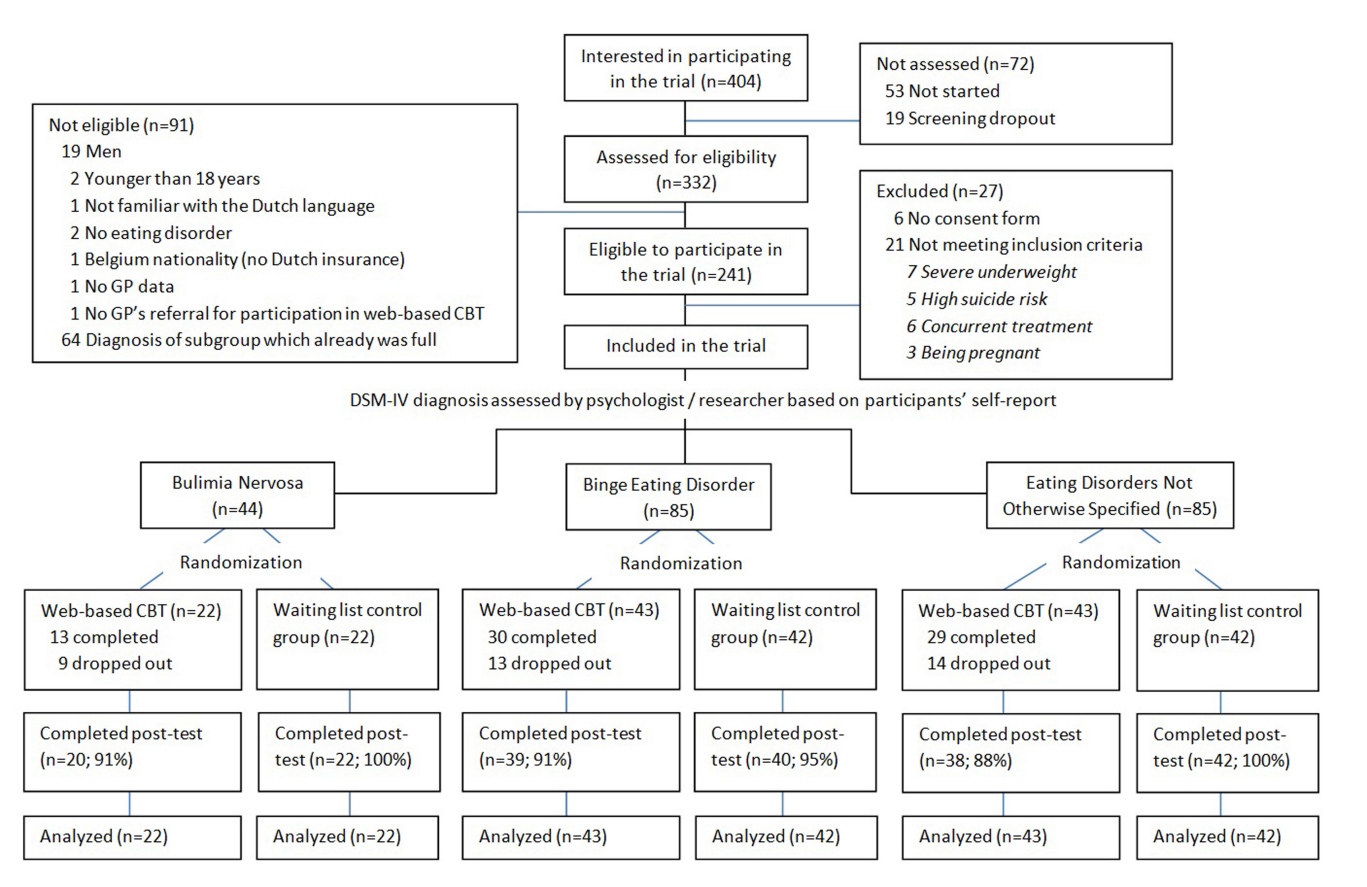
Flow chart of study participants.

From the 404 subjects initially interested in participating in the trial, a total of 214 participants were randomized to one of the two conditions (Web-based CBT or WL), stratified by type of eating disorder (subgroups BN, BED, EDNOS). As shown in Figure 4, the predetermined sample size of 84 participants per eating disorder subtype had not been reached for the subtype BN (n=44). Within the Web-based CBT group, a total of 72 participants (66.7%, 72/108) completed the intervention and 36 participants (33.3%, 36/108) were considered treatment non-completers. Posttest assignments were completed for 201 participants (93.9%, 201/214) with a higher study dropout in the Web-based CBT (10.2%, 11/108) than WL (1.9%, 2/106) (*χ* ^2^_1_=6.46, *P*=.01). Participants who withdrew from the study more often lived alone (*χ* ^2^_1_=5.74, *P*=.04) and had less self-esteem (*t*_212_=2.53, *P*=.01) at baseline than participants who completed the posttest. Within the Web-based CBT group, 99% of the treatment completers (71/72) and 56% of the treatment non-completers (20/36) completed the questions regarding treatment acceptability. Reasons for treatment non-completion were given by 67% of the non-completers (24/36).

### Participant Characteristics

The baseline characteristics of the participants are presented in Table 1. Participants were 214 females with an average age of 39 years, mostly living with others (74.3%, 159/214), employed (79.0%, 169/214), higher educated (50.9%, 109/214) and being overweight (85.0% BMI >25, 182/214). For most participants, the history of the eating disorder was long (68.2% >10 years, 146/214), whereas only 44.4% (95/214) received prior care for their disorder.

The three subgroups BN, BED, and EDNOS differed significantly in terms of age, living situation, and daily routine (data presented in Table 1). BN participants were younger, lived more often alone, and less frequently had a regular daily routine. Also the illness-related variables differed significantly between the three groups, except for the quality of life. Eating disorder psychopathology and mental health were most severe within the BN subgroup and least severe within the EDNOS subgroup. BN participants also had a significantly lower BMI and more problems in social functioning than the other two groups, and significantly more physical complaints and less self-esteem than the EDNOS participants. Participants of the BED subgroup were significantly less satisfied with their body than participants of the EDNOS subgroup.

Baseline characteristics showed no significant differences between the Web-based CBT (n=108) and WL (n=106), nor between these two groups within the three subgroups (data not shown), with the exception of a higher EDE-Q subscale “Restraint” in the EDNOS Web-based CBT subgroup (*t*_83_=2.05, *P*=.04).

**Table 1:** Baseline characteristics of participants.

Variable		Overall (n=214)	BN (n=44)	BED (n=85)	EDNOS (n=85)	Test statistic	df	*P* value
Age (years), mean (SD)		39.4 (11.6)	33.2 (10.4)	40.2 (11.4)	41.9 (11.3)	9.00	2	<.001^hi^
**Living situation, n (%)**						7.93	2	.02
	Alone	55 (25.7)	17 (39)	24 (28)	14 (16)			
	With others	159 (74.3)	27 (61)	61 (72)	71 (84)			
**Level of education, n (%)**						3.59	4	.47
	Low	25 (11.7)	2 (4)	13 (15)	10 (12)			
	Intermediate	80 (37.4)	19 (43)	31 (36)	30 (35)			
	High	109 (50.9)	23 (52)	41 (48)	45 (53)			
Employed, n (%)		169 (79.0)	33 (75)	66 (78)	70 (82)	1.09	2	.58
Regular daily routine, n (%)		156 (72.9)	24 (55)	60 (71)	72 (85)	13.73	2	.001
Prior eating disorder treatment, n (%)		95 (44.4)	17 (39)	39 (46)	39 (46)	0.74	2	.69
Prior psychological treatment, n (%)		143 (66.8)	35 (80)	58 (68)	50 (59)	5.74	2	.06
**Body Mass Index, n (%)**						67.01	4	<.001
	<18.5	6 (2.8)	3 (7)	-	3 (4)			
	18.5-25	26 (12.1)	20 (46)	-	6 (7)			
	>25	182 (85.0)	21 (48)	85 (100)	76 (89)			
**Duration of eating disorder, n (%)**						11.99	6	.06
	1-5 years	33 (15.4)	10 (23)	7 (8)	16 (19)			
	6-10 years	35 (16.4)	8 (18)	17 (20)	10 (12)			
	11-20 years	64 (29.9)	12 (27)	32 (38)	20 (24)			
	>20 years	82 (38.3)	14 (32)	29 (34)	39 (46)			
**Eating disorder psychopathology** ^a^ **, mean (SD)**		3.5 (0.9)	4.1 (0.9)	3.6 (0.8)	3.0 (0.9)	23.54	2	<.001^hij^
	Restraint	2.6 (1.3)	3.5 (1.1)	2.4 (1.3)	2.3 (1.3)	15.00	2	<.001^hi^
	Eating concern	2.9 (1.3)	3.5 (1.1)	3.3 (1.2)	2.2 (1.3)	23.39	2	<.001^ij^
	Shape concern	4.4 (1.1)	4.8 (1.1)	4.6 (0.9)	3.9 (1.1)	13.53	2	<.001^ij^
	Weight concern	4.1 (1.1)	4.5 (1.2)	4.2 (0.8)	3.7 (1.1)	7.95	2	.001^ij^
Body dissatisfaction^b^, mean (SD)		59.3 (14.8)	59.9 (17.4)	62.6 (14.1)	55.7 (13.5)	4.71	2	.01^j^
Physical health^c^, mean (SD)		24.2 (12.3)	28.9 (13.4)	24.3 (11.3)	21.7 (12.1)	5.16	2	.006^i^
MAP-HSS, mean (SD)		12.3 (6.2)	13.1 (6.7)	12.7 (5.8)	11.5 (6.3)	1.31	2	.27
**Mental health** ^d^ **, mean (SD)**		33.1 (19.4)	43.0 (20.7)	33.5 (18.1)	27.6 (18.0)	19.83	3	<.001^hij^
	Depression	11.4 (8.4)	14.9 (8.7)	11.6 (7.9)	9.5 (8.2)	13.82	3	.001^hi^
	Anxiety	6.0 (6.4)	8.5 (7.4)	6.3 (6.5)	4.5 (5.3)	10.85	3	.004^i^
	Stress	15.7 (8.6)	19.7 (9.0)	15.7 (8.3)	13.6 (8.0)	7.78	2	.001^hi^
Self-esteem^e^, mean (SD)		16.7 (7.2)	14.1 (7.4)	16.4 (6.6)	18.4 (7.2)	5.57	2	.004^i^
Quality of life^f^, mean (SD)		62.0 (17.1)	61.0 (17.2)	60.1 (15.6)	64.5 (18.4)	1.50	2	.23
Social functioning^g^, mean (SD)		6.7 (4.8)	8.8 (5.5)	6.6 (4.6)	5.7 (4.4)	6.21	2	.002^hi^

^a^Eating Disorder Examination-Questionnaire.

^b^Body Attitude Test.

^c^Total score of Maudsley Addiction Profile-Health Symptom Scale (MAP-HSS) and 15 additional eating disorder-specific physical complaints.

^d^Depression Anxiety Stress Scale.

^e^Rosenberg Self-Esteem Scale.

^f^EuroQol visual analogue scale.

^g^Measurements in the Addictions for Triage and Evaluation – International Classification of Functioning, Disability and Health.

^h^Significant difference between BN and BED.

^i^Significant difference between BN and EDNOS.

^j^Significant difference between BED and EDNOS.

### Efficacy of Web-Based Treatment for Eating Disorders in General

Table 2 reports the primary and secondary treatment outcomes of this study for all participants at posttest. Participants of the Web-based CBT improved significantly more on eating disorder psychopathology than participants of the WL, although both groups showed improvements over time. Web-based CBT participants also had significantly fewer concerns about their eating, shape, and weight after participating in the intervention than participants of the WL.

Body dissatisfaction, physical health, mental health, self-esteem, and social functioning all improved significantly more in participants of the Web-based CBT than in participants of the WL. Therefore, participating in the intervention resulted in an overall improvement on health indicators related to eating disorders, although effect sizes were generally small. For BMI, no significant effects were found in either group.

During the intervention or waiting period, several participants in both groups had other support for their eating disorder, for example, from family or friends, or through a self-help program (Web-based CBT 24%, 23/96; WL 30.8%, 32/104). Some of these participants had face-to-face contact with a professional such as a therapist, dietician, or GP (Web-based CBT 11%, 11/96 and WL 18.3%, 19/104). For the Web-based CBT group, the additional support had no added value as there were no significant differences between participants who had other (professional face-to-face) support and those who did not have this support during the intervention period. For the WL, additional support had only a significant effect on body dissatisfaction in case of face-to-face contact with a professional. Participants who had face-to-face contact with a professional during the waiting time improved significantly more on body dissatisfaction than participants who did not have this kind of support (*F*_102_=5.16; *P*=.025). For all other outcome measures, no significant effects were found between those two WL groups. Overall effects of the Web-based CBT intervention did also not change significantly by correcting for additional face-to-face support (data not shown). As we used an intention-to-treat (ITT) analysis, we therefore did not correct for this.

**Table 2:** Treatment outcomes for all participants and for the individual subgroups BN, BED, and EDNOS.

			Web-based CBT (n=108)				WL (n=106)				Interaction effect of group x time^a^			
			Baseline	Posttest	*P*	*d*	Baseline	Posttest	*P*	*d*	F	df	*P*	*d*
**All participants (n=214)**														
	**Eating disorder psychopathology** ^b^		3.5 (0.9)	2.6 (1.2)	<.001	.82	3.5 (1.0)	3.0 (1.1)	<.001	.43	9.42	201	.002	.44
		Restraint	2.7 (1.3)	2.2 (1.4)	<.001	.39	2.5 (1.4)	2.1 (1.4)	.046	.23	0.90	206	.34	.15
		Eating concern	2.8 (1.2)	1.7 (1.3)	<.001	.83	3.0 (1.4)	2.4 (1.5)	<.001	.40	6.65	205	.01	.35
		Shape concern	4.4 (1.1)	3.4 (1.5)	<.001	.72	4.4 (1.1)	3.9 (1.3)	<.001	.38	7.87	200	.006	.43
		Weight concern	4.0 (1.1)	3.1 (1.4)	<.001	.73	4.1 (1.0)	3.7 (1.2)	<.001	.36	11.13	200	.001	.48
	Body dissatisfaction^c^		58.4 (14.9)	50.5 (17.6)	<.001	.49	60.3 (14.8)	58.6 (15.3)	.05	.11	13.16	201	<.001	.42
	**Body Mass Index**													
		<18.5	17.2 (0.3)	17.2 (0.6)	.93	.03	17.9 (-)	17.9 (-)	-	-	<0.01	4	.98	.04
		18.5 – 25	22.4 (1.7)	23.2 (1.2)	.09	.58	22.0 (1.7)	22.3 (2.5)	.56	.10	1.43	23	.24	.38
		>25	33.5 (5.7)	33.3 (5.6)	.23	.04	34.0 (5.4)	34.0 (5.3)	.93	.00	0.94	169	.33	.04
	**Physical health** ^d^		22.8 (12.7)	18.1 (12.7)	<.001	.37	25.7 (11.8)	24.3 (12.2)	.045	.11	12.55	200	<.001	.28
		MAP-HSS	11.7 (6.5)	9.5 (6.4)	<.001	.34	13.0 (5.9)	12.2 (5.9)	.046	.13	6.94	202	.009	.23
	**Mental health** ^e^		31.7 (18.6)	23.6 (18.5)	<.001	.43	34.6 (20.2)	31.2 (20.7)	.03	.17	4.88	203	.03	.24
		Depression	11.2 (8.4)	7.5 (7.5)	<.001	.46	11.7 (8.5)	10.2 (8.6)	.06	.17	4.37	204	.04	.26
		Anxiety	5.5 (5.8)	4.7 (5.4)	.06	.16	6.5 (7.0)	6.4 (6.4)	.84	.02	1.09	206	.30	.12
		Stress	15.0 (8.5)	11.3 (8.4)	<.001	.43	16.4 (8.6)	14.6 (9.0)	.009	.20	3.55	203	.06	.22
	Self-esteem^f^		16.2 (7.1)	18.6 (6.8)	<.001	.34	17.3 (7.2)	18.3 (6.9)	.01	.14	5.06	202	.03	.20
	Quality of life^g^		62.8 (17.2)	69.2 (15.8)	<.001	.39	61.2 (17.1)	65.4 (15.0)	.03	.27	0.85	206	.36	.13
	Social functioning^h^		6.8 (5.0)	5.2 (4.3)	<.001	.35	6.5 (4.7)	6.3 (4.3)	.50	.05	7.93	205	.005	.29
**BN subgroup (n=44)**														
	**Eating disorder psychopathology** ^b^		3.9 (1.0)	2.9. (1.1)	.003	.94	4.2 (0.8)	3.7 (1.1)	.02	.55	1.92	41	.17	.55
		Restraint	3.3 (1.3)	2.6 (1.2)	.01	.64	3.7 (1.0)	3.4 (1.2)	.43	.27	1.11	42	.30	.44
		Eating concern	3.3 (1.1)	1.9 (1.1)	<.001	1.30	3.7 (1.1)	3.0 (1.5)	.04	.53	2.59	41	.12	.65
		Shape concern	4.7 (1.2)	3.8 (1.5)	.02	.67	4.9 (1.0)	4.4 (1.3)	.02	.49	0.74	41	.39	.31
		Weight concern	4.4 (1.3)	3.5 (1.6)	.03	.62	4.7 (1.2)	4.2 (1.2)	.02	.43	0.86	41	.36	.32
**BED subgroup (n=85)**														
	**Eating disorder psychopathology** ^b^		3.5 (0.8)	2.6 (1.3)	<.001	.87	3.7 (0.7)	3.2 (0.9)	.002	.60	4.25	78	.04	.61
		Restraint	2.5 (1.4)	1.9 (1.5)	.005	.42	2.3 (1.3)	2.0 (1.3)	.31	.23	0.60	80	.44	.22
		Eating concern	3.1 (1.2)	2.0 (1.4)	<.001	.86	3.4 (1.1)	2.7 (1.4)	.001	.61	1.36	80	.25	.31
		Shape concern	4.5 (1.0)	3.5 (1.6)	<.001	.76	4.7 (0.8)	4.2 (1.1)	.004	.52	4.13	77	.046	.60
		Weight concern	4.1 (0.9)	3.1 (1.4)	<.001	.83	4.3 (0.8)	3.9 (0.9)	.02	.39	6.67	78	.01	.77
**EDNOS subgroup (n=85)**														
	**Eating disorder psychopathology** ^b^		3.2 (0.8)	2.5 (1.1)	<.001	.76	2.9 (0.9)	2.5 (1.0)	.002	.39	3.31	78	.07	.38
		Restraint	2.6 (1.2)	2.3 (1.3)	.16	.26	2.0 (1.3)	1.7 (1.2)	.11	.28	0.02	80	.90	.03
		Eating concern	2.2 (1.1)	1.4 (1.2)	<.001	.70	2.2 (1.5)	1.8 (1.4)	.04	.25	3.41	79	.07	.35
		Shape concern	4.1 (1.0)	3.2 (1.4)	<.001	.72	3.8 (1.1)	3.4 (1.4)	.02	.32	3.27	78	.07	.44
		Weight concern	3.8 (1.1)	3.0 (1.3)	<.001	.73	3.6 (1.0)	3.2 (1.2)	.005	.37	5.01	78	.03	.44

^a^Treatment outcomes were measured with repeated measures and mixed model analysis. Effect sizes were measured with Cohen’s *d.*

^b^Eating Disorder Examination-Questionnaire.

^c^Body Attitude Test.

^d^Total score of Maudsley Addiction Profile-Health Symptom Scale (MAP-HSS) and 15 additional eating disorder-specific physical complaints.

^e^Depression Anxiety Stress Scale.

^f^Rosenberg Self-Esteem Scale.

^g^EuroQol visual analogue scale.

^h^Measurements in the Addictions for Triage and Evaluation – International Classification of Functioning, Disability and Health.

### Efficacy of Web-Based Treatment for Specific Eating Disorder Subgroups

Among participants of the Web-based CBT, eating disorder psychopathology significantly improved over time for all subgroups (data presented in Table 2). However, participants in the control group also improved, and significant differences in effects between the Web-based CBT and WL on primary outcome were found only in the BED subgroup.

On secondary outcome measures, Web-based CBT participants in all three subgroups improved significantly over time with regard to physical health, mental health, self-esteem, quality of life, and social functioning, with small to moderate effect sizes (data presented in Multimedia Appendix 3). Furthermore, BED and EDNOS participants of the Web-based CBT group also improved significantly regarding body dissatisfaction. In the WL group, no significant time effects were found on the secondary outcome measures with the exception of self-esteem for participants with BN and body dissatisfaction and mental health for participants with EDNOS. However, significant between-group differences (Web-based CBT and WL) within the three subgroups were found only for body dissatisfaction and physical health in BED participants and for body dissatisfaction and mental health in EDNOS participants.

### Treatment Acceptability

In general, participants were satisfied with the Web-based CBT intervention and their therapist. Most participants evaluated the intervention as rather (46%, 42/91) or very (35%, 32/91) useful, and according to the participants the intervention was especially effective for their eating behavior. Four out of five participants (79%, 72/91) indicated that the treatment had resulted in a healthier diet in their daily lives. Furthermore, for several participants the treatment also led to improvement of mental health (56%, 51/91), self-esteem (49%, 45/91), physical health (47%, 43/91), body image (46%, 42/91), and exercise habits (45%, 41/91). On a scale from 0-10, participants evaluated the intervention with a 7.6 (SD 1.3) and their therapist with an 8.1 (SD 1.0). The majority of participants considered the online contact to be (very) pleasant (77%, 70/91), personal (60%, 55/91), and safe (92%, 84/91). Almost all participants evaluated the support of the therapist to be of added value. For participants who completed the intervention, the therapeutic support was one of the most valuable and important components of the treatment. However, some participants had missed other forms of contact (eg, face-to-face or via telephone) a little (33%, 30/91), quite a lot (5%, 5/91), or very much (8%, 7/91). The participants who did not complete the intervention often mentioned several reasons for dropping out or stopping the intervention prematurely, which can be divided into three main categories: (1) personal reasons or problems (33%, 8/24; eg, lack of time, psychological problems, lack of motivation), treatment content/protocol (29%, 7/24; eg, eating diary annoying/too time consuming, assignments not supportive, not enough attention for weight loss), and the online method (21%, 5/24; eg, lack of personal contact, too open-ended). Furthermore, two participants were discharged by the therapist due to the seriousness of their problems and referred to a more appropriate treatment, and one participant stopped because her GP and psychologist considered the intervention not suitable and had reported her to an outpatient mental health facility for face-to-face treatment.

## Discussion

### Principal Results and Comparison With Prior Work

Our study shows that Web-based CBT is effective in reducing eating disorder psychopathology in participants with eating disorders in comparison with a waiting list control group. This finding is consistent with the results of a recent review on Internet-based treatments of eating disorders [10]. Participants of the Web-based CBT reported significant reductions in eating disorder psychopathology and were also less concerned with their eating, shape, and weight after participating in the intervention. Participation in the Web-based CBT also resulted in a significant reduction in body dissatisfaction and an improvement of physical and mental health, self-esteem, and social functioning. However, participants of the control group also improved on almost all eating disorder and health-related outcomes resulting in small to moderate effect sizes for the Web-based CBT on interaction effects. The reasons for the improvements in the control group are not totally clear, but several participants received other professional face-to-face support during the waiting period and this resulted in an improvement of participants’ body dissatisfaction. Furthermore, it is possible that the no-reply email messages that participants received during the waiting period activated them to start behavioral change. Possibly the waiting list condition thus better resembled an unguided self-help condition than a no-intervention condition. Additionally, the process of seeking help or knowing that treatment would start shortly could also have contributed to the improvement, as some other studies showed similar results [39,40].

For BMI, no effects were found. Most participants were overweight at baseline and therefore improvement of BMI would be desirable. It would be interesting to use the follow-up data to investigate whether the BMI of participants will improve in the long term.

In comparison to the results of our pilot study [18], similar significant effects were found, but with somewhat lower effect sizes for the Web-based CBT. For eating disorder psychopathology, the effect size was *d*=1.14 in the pilot study and *d*=.82 in the current trial. This difference is most likely because the pilot results included only posttest data of participants who had completed the intervention, whereas the data of this study also included posttest data of treatment non-completers (33% of Web-based CBT group). As the content and protocol of the intervention, and the online method were important reasons for dropping out or stopping the intervention prematurely, it is likely that the results of the intervention are less positive for treatment non-completers than for treatment completers. For future research, it would be interesting to compare the results of treatment completers and treatment non-completers in terms of efficacy and acceptability of the intervention.

The treatment adherence of 67% in this study was remarkably higher than in our pilot study (54%) [18]. A possible explanation could be that the higher threshold to participate in the current study (because of the randomized design, GP referral, and informed consent) has resulted in selection bias. It is rather difficult to compare the treatment adherence of our Web-based CBT intervention to the adherence of other Web-based interventions, as the definition of adherence is quite diverse in the different studies [10]. However, based on the results of a systematic review on other Web-based interventions for patients with eating disorders [10], the compliance rate of 67% with participants completing all treatment modules of the intervention, can be considered as moderate to high. Compared to the compliance rate of 50% found in a systematic review on adherence in Web-based interventions focused on broader health issues [41], the treatment adherence in our study was significantly higher.

In addition to other studies, we compared the effects of the Web-based CBT between patients with different eating disorder diagnoses (BN, BED, and EDNOS). Web-based CBT was primarily effective for participants with BED. For this subgroup, significant interaction effects were found for eating disorder psychopathology, body dissatisfaction, and mental health, with medium effect sizes. According to the results of a recent review [10], the improvement in eating disorder psychopathology among BED participants in our study is similar to the results found for participants with BED participating in a 6-month self-help treatment with weekly therapist support [42]. For the EDNOS subgroup, participating in the Web-based CBT did not result in a significant interaction effect on eating disorder psychopathology, although the within-group effect size was rather high (*P*<.001, *d*=.71). As participants’ body dissatisfaction and mental health did improve significantly, and also high within-group effects were found for the other outcome measures, the intervention was partly effective for this subgroup as well. Because EDNOS is not a homogeneous group, further research should elucidate whether the intervention may be more or less effective for specific subgroups of patients with an EDNOS diagnosis. For example, it would be interesting to investigate the results of the intervention among the participants of the EDNOS category who did meet a specified eating disorder diagnosis using the DSM-5 criteria. This was applicable for 29% of all EDNOS participants since 11 participants of the Web-based CBT group (26%) and 8 participants of the WL group (19%) met the DSM-5 criteria of BED, 2 participants of the Web-based CBT group (5%) met the DSM-5 criteria of AN, and 3 participants of the WL group (7%) met the DSM-5 criteria of BN. Though participants with BN improved during treatment with a high within-group effect size for eating disorder psychopathology and small to moderate within-group effect sizes for most secondary outcome measures, no significant interaction effects were found for any outcome measure. This could be explained by the smaller sample size of this subgroup (n=44). However, other explanations are possible, for example, that participants with BN need a more intensive (face-to-face) treatment as results showed that these subgroups had more severe eating disorder psychopathology and related health problems at baseline than the other subgroups. Therefore, it would be interesting to further evaluate the results of the intervention for this specific subgroup.

Participants were generally satisfied with the Web-based CBT intervention, and the support of the therapist was considered as very valuable and important. Several methods were used to ensure quality and consistency in the treatment of participants (eg, formats for each intervention module, intensive training, and supervision of therapists, and retrospective control of therapists’ messages). Therefore we expect only minor differences in the support provided by the therapists, equivalent to differences between therapists in clinical face-to-face treatment. However, differences in therapeutic support possibly resulted in differences between therapists in participant outcome. As the 17 therapists were not stratified by type of eating disorder, and the number of participants assigned to each therapist differed significantly with a minimum of 2 and a maximum of 31 participants, this might have affected the results of the intervention. Therefore, it would be interesting to investigate the consistency in the support of the therapists, their adherence to the protocol, and their competencies, for example, by conducting a study including directive and summative content analysis of the treatment of several participants of the current study. Furthermore, additional research into the differences between therapists in participant outcome would be very valuable.

Because the effectiveness of the Web-based CBT was investigated within a naturalistic setting, results of this study are likely to approximate those of the Web-based intervention in everyday practice. Another strength of our study is the low study dropout, as posttest data were available for 94% of the participants.

### Limitations

Although the study shows promising results, these should be considered in the context of several limitations. The first and most important limitation is that, despite extending the sampling period, we were unable to recruit the sample size of 84 participants with BN and therefore also did not reach the planned total sample size of 252 participants. Although the total sample size of 252 participants was not achieved, significant time and interaction effects for the overall group were found for almost all outcome measures. For participants with BN on the other hand, only significant time effects were found; there were no significant interaction effects.

Second, the number of participants per diagnostic category was low. Sample sizes were calculated based on the results of our pilot study. However, we did not sufficiently take into account the differences in effectiveness between the subgroups and the improvements among participants of the control group, resulting in a low number of participants per diagnostic category.

Third, eating disorder diagnoses were not assessed using an in-person clinical interview as required in formal diagnoses, but by using online self-report questions including the EDQ-O. As an in-person interview would probably increase the threshold to participate in the Web-based CBT intervention, we found this incompatible with the main objective of this treatment. Therefore, we decided to use only self-report assessments to measure all primary and secondary outcomes. A recent study [35] showed acceptable performance of the EDQ-O as a diagnostic instrument for all DSM-IV eating disorder classifications, although improvement was desirable. However, this study did not use the EDQ-O as the only tool to assess eating disorder diagnoses. Also, other baseline data were taken into consideration, and if necessary, additional questions were asked by email. Nevertheless, the lack of any personal interviews is an important limitation of this study. For future research, this topic should be considered carefully, weighing pros (validity) and cons (excluding patients).

### Conclusions

Eating disorders have a considerable impact on the quality of life and psychological and physical health of patients. The participants in this study suffered from BN, BED, and EDNOS for several years, and more than half of the participants had never had treatment for their eating disorder. The results of this study provide support for the use of a Web-based CBT intervention to improve eating disorder psychopathology, body dissatisfaction, and related health among patients with eating disorders. Furthermore, our findings confirm that new technologies can be used to treat patients who otherwise refrain from seeking or receiving help.

## Appendix

Multimedia Appendix 1 CONSORT-EHEALTH checklist V1.6.2 [42].

Multimedia Appendix 2 Content of Web-based CBT intervention.

Multimedia Appendix 3 Treatment results on secondary outcome measures for participants of the subgroups BN, BED, and EDNOS.

## Abbreviations

BAT: Body Attitude Test
BED: binge-eating disorder
BMI: body mass index
BN: bulimia nervosa
CBT: cognitive behavioral therapy
DASS: Depression Anxiety Stress Scale
DSM-IV: Diagnostic and Statistical Manual of Mental Disorders, 4th revision
EDE-Q: Eating Disorder Examination-Questionnaire
EDNOS: eating disorder not otherwise specified
EDQ-O: Eating Disorder Questionnaire-Online
EQ-5D VAS: EuroQol-5D visual analogue scale
GP: general practitioner
MAP-HSS: Maudsley Addiction Profile-Health Symptom Scale
MATE-ICN: Measurements in the Addictions for Triage and Evaluation – International Classification of Functioning, Disability and Health
MINI-Plus: Mini International Neuropsychiatric Interview-Plus
NTR: Nederlands Trial register
RCT: randomized controlled trial
RSES: Rosenberg Self-Esteem Scale
