# Supplementary material for: Web-Based Cognitive Behavioral Therapy for Female Patients With Eating Disorders: Randomized Controlled Trial
Source: J Med Internet Res. 2015 Jun 18;17(6):e152. doi: 10.2196/jmir.3946 (PMC4526949; doi:10.2196/jmir.3946)
Supplement: Multimedia Appendix 1 [file jmir_v17i6e152_app1.pdf]

CONSORT-EHEALTH Checklist V1.6.2 Report

(based on CONSORT-EHEALTH V1.6), available at [http://tinyurl.com/consort-ehealth-v1-6].

3946

Date completed

10/14/2014 7:43:22

by

Elke D. ter Huurne

Web-based Cognitive Behavioral Therapy for female patients with bulimia nervosa, binge eating disorder, and eating disorders not otherwise specified: a Randomized Controlled Trial.

TITLE

1a-i) Identify the mode of delivery in the title

Yes: "Web-based Cognitive Behavioral Therapy ..."

1a-ii) Non-web-based components or important co-interventions in title

This is not included in the title because the study did not use other forms of intervention.

1a-iii) Primary condition or target group in the title

Yes: "Web-based Cognitive Behavioral Therapy for female patients with bulimia nervosa, binge eating disorder, and eating disorders not otherwise specified: a Randomized Controlled Trial."

ABSTRACT

1b-i) Key features/functionalities/components of the intervention and comparator in the METHODS section of the ABSTRACT

Yes: "This study evaluated the effects of a web-based cognitive behavioral therapy (CBT) intervention using intensive asynchronous therapeutic support to improve eating disorder psychopathology and to reduce body dissatisfaction and related health problems among patients with eating disorders."

1b-ii) Level of human involvement in the METHODS section of the ABSTRACT

Yes: "Participants had asynchronous contact with a personal therapist twice a week, solely via the internet."

1b-iii) Open vs. closed, web-based (self-assessment) vs. face-to-face assessments in the METHODS section of the ABSTRACT

Yes: "Participants were recruited from an open access website ..." and "Participants had asynchronous contact with a personal therapist twice a week, solely via the internet." and "Self-report measures of eating disorder psychopathology (primary outcome), body dissatisfaction, physical health, mental health, self-esteem, quality of life, and social functioning were completed at baseline and post-test."

1b-iv) RESULTS section in abstract must contain use data

Yes: "A total of 214 participants were randomized to either the web-based CBT group (n=108) or to the WL group (n=106) stratified by type of eating disorder (BN: n=44; BED: n=85; EDNOS: n=85). Study attrition was low with 94% of the participants completing the post-test assignment. For the overall group, web-based CBT showed a significant improvement over time for eating disorder psychopathology (F=63.07, df=97, p<.001, d=.82) and all secondary outcome measures (effect sizes between d=.34 to d=.49), except for BMI. WL participants also improved on most outcomes; however, effects were smaller in this group with significant between-group effects for eating disorder psychopathology (F=9.42, df=201, p=.002, d=.44), body dissatisfaction (F=13.16, df=201, p<.001, d=.42), physical health (F=12.55, df=200, p<.001, d=.28), mental health (F=4.88, df=203, p=.028, d=.24), self-esteem (F=5.06, df=202, p=.026, d=.20) and social functioning (F=7.93, df=205, p=.005, d=.29). Analyses for the individual subgroups BN, BED, and EDNOS showed that eating disorder psychopathology improved significantly over time among web-based CBT participants in all three subgroups; however, the between-group effect was only significant for participants with BED (F=4.25, df=78, p=.043, d=.61)."

1b-v) CONCLUSIONS/DISCUSSION in abstract for negative trials

Yes: "Web-based CBT proved to be effective in improving eating disorder psychopathology and related health among female patients with eating disorders."

INTRODUCTION

2a-i) Problem and the type of system/solution

Yes: "In the Netherlands, eating disorders have a lifetime prevalence of 1.74% [1] and these disorders account for severe psychological, physical and social morbidity. Although early identification and treatment is desired, patients often refrain from seeking or receiving help because of personal barriers, such as feelings of shame and fear of stigmatization, and intervention-related barriers, such as costs, geographical distance and lack of availability [2-6]. Psychiatric services are challenged to help patients overcome these barriers by providing easily accessible, low-threshold interventions. The internet offers many possibilities for these types of interventions because of its relative anonymity, widespread and 24-hour access, and increasing usage. ….. In the past few years several internet interventions have been developed for patients with eating disorders, and a recent review showed that these treatments can be effective in reducing eating disorder psychopathology, binge eating and purging, as well as in improving quality of life [10]. However, it should be noted that most studies were conducted among patients with bulimia nervosa (BN) and (to a lesser extent) binge eating disorder (BED) [10], whereas eating disorders not otherwise specified (EDNOS) is the most commonly diagnosed eating disorder [11]. Therefore, in 2009 a web-based CBT intervention with intensive therapeutic support was developed for Dutch patients with all types of eating disorders [12], …"

2a-ii) Scientific background, rationale: What is known about the (type of) system

Yes: "In 2010, a before-after study into this intervention showed a reduction in eating disorder psychopathology (d=1.14) and body dissatisfaction (d=0.86), as well as high patient satisfaction [15]. However, this study had a nonrandomized design and only included those participants who completed the intervention (54% of participants)."

METHODS

3a) CONSORT: Description of trial design (such as parallel, factorial) including allocation ratio

Yes: The present study, therefore, aimed to explore the effects of this web-based CBT intervention, compared to a waiting list control group (WL), on eating disorder psychopathology (primary outcome) as well as body dissatisfaction, physical health, BMI, mental health, self-esteem, quality of life, and social functioning (secondary outcomes). Furthermore, we were interested in the effects of the web-based CBT across participants of the specific eating disorder subgroups: BN, BED, and EDNOS."

3b) CONSORT: Important changes to methods after trial commencement (such as eligibility criteria), with reasons

This item is not applicable to this study.

3b-i) Bug fixes, Downtimes, Content Changes

This item is not applicable to this study.

4a) CONSORT: Eligibility criteria for participants

Yes. In 'Participants' we report that "Inclusion criteria for participation were: (1) female gender; (2) age ≥ 18 years; (3) diagnosis of BN, BED, or EDNOS; (4) written and oral fluency in Dutch language; (5) access to internet; (6) signed informed consent; and (7) a GP referral. Exclusion criteria were: (1) severe underweight; (2) suicidal ideation; (3) receiving psychological or pharmaceutical treatment for any eating disorder within the past six months; (4) pregnancy; and (5) ≥ 4 weeks absence during the intervention period. If participation in the intervention was not possible for some reason (e.g., lack of Dutch health insurance and therefore funding of the intervention, or patient's GP did not agree with participation), patients were also not eligible for this study."

4a-i) Computer / Internet literacy

Yes. In ‘Participants’ we mention that “Inclusion criteria for participation were: ..…(4) written and oral fluency in Dutch language; (5) access to internet; ….”. We did not explicitly check the computer/internet literacy. However, the registration process acted as in implicit computer / internet literacy test. If participants were able to complete the registration, they were sufficiently equipped to participate in the web-based intervention.

4a-ii) Open vs. closed, web-based vs. face-to-face assessments:
Yes. In ‘Participants we report that “Participants were self-recruited users of the Dutch website ‘Look at your eating’ (www.etendebaas.nl) [16]. This open access website offered general information on eating disorders and related topics, a forum for peer support, as well as information about the web-based CBT program and the study procedures of this trial. “, in ‘Study design and procedure’ we report that “Information about the study was disseminated through announcements on eating disorder-related websites and forums, and newspaper advertisements.” and in ‘Intervention’ we mention that “During the web-based CBT participants normally had asynchronous contact twice a week with their personal therapists, solely via the internet, unless participants specifically requested an additional telephone contact.”

4a-iii) Information giving during recruitment
Yes. In ‘Study design and procedure’ we report that “Information about the study was disseminated through announcements on eating disorder-related websites and forums, and newspaper advertisements. Website visitors were invited to read the information about the study explicitly, provide their e-mail addresses and telephone numbers, and agree with the conditions of the web-based CBT protocol. Furthermore, they had to provide written informed consent, personal data and data of their general practitioner (GP).”
We did not publish the informed consent documentation as appendix, because the information is written in the Dutch language and therefore not readable for many readers of the Journal of Medical Internet Research.

4b) CONSORT: Settings and locations where the data were collected
Yes. In ‘Measures’ we report that “Participants completed these online self-report measures via their personal file within the web-based application at baseline and post-test (approximately 15 weeks after baseline).“

4b-i) Report if outcomes were (self-)assessed through online questionnaires
Yes. In ‘Measures’ we clarify that “Participants completed these online self-report measures via their personal file within the web-based application at baseline and post-test (approximately 15 weeks after baseline).“

4b-ii) Report how institutional affiliations are displayed

This item is not applicable to this study.

5) CONSORT: Describe the interventions for each group with sufficient details to allow replication, including how and when they were actually administered

5-i) Mention names, credential, affiliations of the developers, sponsors, and owners
Yes. In ‘Intervention’ we report that “The web-based CBT intervention [12, 15] called “Look at your eating” (in Dutch: Etendebaas.nl) was developed at Tactus Addiction Treatment by health care professionals (social workers, registered nurses, a dietician, a psychologist, a doctor specialized in addiction, a psychotherapist and a psychiatrist), a software development team (The Factor-E), and patients and members of a Dutch national patient organization for persons with eating disorders and their social environment.”
Furthermore, in ‘Conflicts of interests’ we mention that “Elke ter Huurne and Hein de Haan participated in the development of the web-based CBT intervention, but they did not derive financial benefit from the intervention. All other authors declare no conflicts of interest.”

5-ii) Describe the history/development process
Yes. In ‘Intervention’ we report that “Development of the intervention was an interactive and iterative process, involving patients providing input and feedback on different versions of the content, layout, visual features, and ease of navigation of the web-based CBT intervention.”

5-iii) Revisions and updating

The content of the web-based CBT intervention was “frozen” during the trial and represents a static intervention.

5-iv) Quality assurance methods
The content of the intervention was based on evidence-based methods (CBT and Motivational Interviewing) and pre-programmed text fragments were used to ensure accuracy and quality of information. Furthermore, Hein de Haan, psychiatrist/MD and second author of this manuscript, has been closely involved in the development of the intervention. All therapists were intensively trained in working with the web-based intervention and during the intervention they also were supervised by coaches and a licensed psychotherapist. This is reported in the ‘Intervention’ section.

5-v) Ensure replicability by publishing the source code, and/or providing screenshots/screen-capture video, and/or providing flowcharts of the algorithms used
In 2013, we have published the results of our pilot study in JMIR (in the current manuscript we also refer to this article) and in that publication we already added some screenshots of the website and the personal file of participants. However, if preferred, we will be happy to add more information and screenshots of the intervention, for example of specific assignments or the online eating diary.

5-vi) Digital preservation
Yes. We included a reference for the web-based CBT intervention: Tactus Addiction Treatment. Web-based CBT intervention Etendebaas.nl. URL:http://www.etendebaas.nl. [Accessed: 2014-10-03] (Archived by WebCite at http://www.webcitation.org/6T2iDWuKN). A demo of the intervention was realized by creating several (YouTube) videos detailing all important aspects of the intervention. These videos are available via the homepage of the website. However, they are only presented in Dutch.

5-vii) Access
Yes. In ‘Intervention’ we mention that “Participants could access the intervention in their personal environment at any time by logging in to their personal file via the website [16], where they could read the messages from their therapist or complete assignments.“ and “For all participants, the intervention was covered by the Dutch health insurance, although the costs were set off against the deductible of the participants.” As described in subitem 5-vi, a Dutch demo is available via the website www.etendebaas.nl.

5-viii) Mode of delivery, features/functionalities/components of the intervention and comparator, and the theoretical framework
Yes: In ‘Intervention’ we report that “The intervention included a structured two-part program with at least 21 contact moments and 10 assignments within a secure web-based application. The first part focused on the analysis of the participant’s eating attitudes and behaviors, and the second part focused on behavioral change. CBT [18-20] and Motivational Interviewing [21, 22] formed the fundamentals of the intervention including techniques such as psycho education, self-monitoring, thought restructuring, problem solving, and relapse prevention. During the web-based CBT participants normally had asynchronous contact twice a week with their personal therapists, solely via the internet, unless participants specifically requested an additional telephone contact. Participation in the intervention took approximately 20 minutes per day and participants were instructed about completing home-assignments and registering eating behaviors daily in their online eating diary. …… Accordingly, therapists responded within 3 working days on the participants’ messages or assignments. The therapists’ messages were personalized to the participants’ situation but also consisted pre-programmed text fragments, e.g., to explain the assignments.”

5-ix) Describe use parameters
Yes. In ‘Intervention’ we mention that “Participation in the intervention took approximately 20 minutes per day and participants were instructed about completing home-assignments and registering eating behaviors daily in their online eating diary.”

5-x) Clarify the level of human involvement
Yes. In ‘Intervention’ we report that “During the web-based CBT participants normally had asynchronous contact twice a week with their personal therapists, solely via the internet, unless participants specifically requested an additional telephone contact.” and “ …. therapists responded within 3 working days on the participants’ messages or assignments.”
Furthermore, we clarify that “Seventeen therapists with either a bachelor’s degree in nursing or social work or a master’s degree in psychology were involved in this study. They all completed a two-day training, a full treatment program with a test patient and three months of intensive supervision by highly experienced coaches. Furthermore, a multidisciplinary team consisting of a psychologist, a psychotherapist, a doctor specialized in addiction, and a psychiatrist gave expert advice at the end of part 1 of the intervention, and they were also available for consultation.”

5-xi) Report any prompts/reminders used

Yes. In ‘Intervention’ we report that “The progress of the intervention was monitored by the therapists. When participants did not respond to the messages of the therapists within the next week, they received a reminder with a request to keep in touch regularly. When participant did not respond for four weeks, the intervention was terminated prematurely.

5-xii) Describe any co-interventions (incl. training/support)

The web-based CBT is a stand-alone intervention; there are no other co-interventions. Therefore, this item is not relevant for this study.

6a) CONSORT: Completely defined pre-specified primary and secondary outcome measures, including how and when they were assessed

Yes: In ‘Measures’ we report that “The primary outcome measure was eating disorder psychopathology measured with the global mean scale of the Eating Disorder Examination-Questionnaire (EDE-Q) [23], a widely used self-report questionnaire to measure eating disorder severity. Items were scored on a seven-point Likert scale (range 0 – 6), with a higher score reflecting more psychopathology. Additionally, the scores on the EDE-Q subscales Restraint, Eating Concern, Shape Concern, and Weight Concern were calculated. Secondary outcome measures included the Body Attitude Test (BAT) [24, 25] to assess body dissatisfaction, the Maudsley Addiction Profile-Health Symptom Scale (MAP-HSS) [26] and 15 eating disorder-specific physical complaints to measure physical health, the Depression Anxiety Stress Scale (DASS) [27] to measure mental health, the Rosenberg Self-Esteem Scale (RSES) [28] to examine self-esteem, the EuroQol visual analogue scale (EQ-5D VAS) [29] to assess quality of life, the Measurements in the Addictions for Triage and Evaluation - International Classification of Functioning, Disability and Health (MATE-ICN) [30] to examine social functioning, and body mass index (BMI). Participants completed these online self-report measures via their personal file within the web-based application at baseline and post-test (approximately 15 weeks after baseline).”

6a-i) Online questionnaires: describe if they were validated for online use and apply CHERRIES items to describe how the questionnaires were designed/deployed

6a-ii) Describe whether and how “use” (including intensity of use/dosage) was defined/measured/monitored

Yes, we report that participants were considered as completers when they completed all 10 assignments and attended at least 21 sessions with their personal therapist (see ‘Participant flow’). Furthermore, in ‘Intervention’ we report that “Participation in the intervention took approximately 20 minutes per day and participants were instructed about completing home-assignments and registering eating behaviors daily in their online eating diary.” and that “The progress of the intervention was monitored by the therapists. When participants did not respond to the messages of the therapists within the next week, they received a reminder with a request to keep in touch regularly. When participants did not respond for four weeks, the intervention was terminated prematurely.”

6a-iii) Describe whether, how, and when qualitative feedback from participants was obtained

6b) CONSORT: Any changes to trial outcomes after the trial commenced, with reasons

The trial outcomes did not change after the trial commenced, so this item is not relevant for the current study.

7a) CONSORT: How sample size was determined

7a-i) Describe whether and how expected attrition was taken into account when calculating the sample size

Yes. In ‘Statistical Analysis’ we report that “Our sample size was calculated based on an expected mean difference score of 1.0 (SD 1.2) on the EDE-Q global score (primary outcome measure) between the web-based CBT and WL at post-test. This expected difference was based on the results of our before-after study, adjusted for an estimated improvement in the WL. Power analysis (G*Power) revealed a sample size of 25 participants in each condition based on a significance level of 5%, a power of 80%, the same number of participants per condition, 2 measurements, and a correlation among repeated measures of 0.95. However, we expected 40% of the participants not to complete the web-based CBT, therefore, 42 participants in each condition (web-based CBT and WL) were needed. To determine the efficacy of the web-based CBT for the specific eating disorder subtypes, the total sample size was determined at 84 participants with BN, 84 participants with BED, and 84 participants with EDNOS (total of 252 participants).”

7b) CONSORT: When applicable, explanation of any interim analyses and stopping guidelines

We did no do interim analyses, but the recruitment of study participants was stopped before the predetermined sample size of 252 participants was reached. We extended the sampling period several times, but were unable to recruit the sample size of 84 participants with BN. Due to lack of time and resources it was not possible to further extend the recruitment of participants and therefore our study includes only 44 participants with BN and subsequently overall 214 participants with eating disorders.

8a) CONSORT: Method used to generate the random allocation sequence

Yes. In ‘Study design and procedure’ we report that “Participants were randomized to the web-based CBT or WL through computer-generated randomly varying block sizes (2, 4 or 8), stratified by type of eating disorder (BN, BED, EDNOS). Randomization was performed at a 1:1 ratio. The allocation schedule was prepared by an independent researcher not involved in data collection.”

8b) CONSORT: Type of randomisation; details of any restriction (such as blocking and block size)

Yes. In ‘Study design and procedure’ we report that “Participants were randomized to the web-based CBT or WL through computer-generated randomly varying block sizes (2, 4 or 8), stratified by type of eating disorder (BN, BED, EDNOS). Randomization was performed at a 1:1 ratio. The allocation schedule was prepared by an independent researcher not involved in data collection.”

9) CONSORT: Mechanism used to implement the random allocation sequence (such as sequentially numbered containers), describing any steps taken to conceal the sequence until interventions were assigned

Yes. In ‘Study design and procedure’ we mention that “The assignment of participants to the conditions was not depending on the participants’ characteristics.”

10) CONSORT: Who generated the random allocation sequence, who enrolled participants, and who assigned participants to interventions

Yes. In ‘Study design and procedure’ we report that “The allocation schedule was prepared by an independent researcher not involved in data collection.” and that “The assignment of participants to the conditions was not depending on the participants’ characteristics.”

11a) CONSORT: Blinding - If done, who was blinded after assignment to interventions (for example, participants, care providers, those assessing outcomes) and how

11a-i) Specify who was blinded, and who wasn’t

Due to the nature of this study, participants as well as therapists and researchers were not blinded to the treatment allocation.

11a-ii) Discuss e.g., whether participants knew which intervention was the “intervention of interest” and which one was the “comparator”

Due to the nature of this study including a waiting list control group, participants knew that the web-based CBT intervention was the “intervention of interest”.

11b) CONSORT: If relevant, description of the similarity of interventions

This is not relevant for this study.

12a) CONSORT: Statistical methods used to compare groups for primary and secondary outcomes

This is not applicable to this study. For statistical methods used for subgroup analyses we refer to subitem 12a-i.

12a-i) Imputation techniques to deal with attrition / missing values

Yes. In ‘Statistical Analysis’ we report “To measure the efficacy of the web-based CBT in terms of primary and secondary outcome measures, Mixed Models for repeated measures were used, allowing for the inclusion of all participants, regardless of missing data.”

12b) CONSORT: Methods for additional analyses, such as subgroup analyses and adjusted analyses

Yes. In ‘Statistical Analysis” we report “Baseline differences between the web-based CBT and WL are expressed as differences in proportion for categorical data and as the mean differences for continuous data. Chi square or Fisher’s exact tests (as appropriate) were used to compare categorical measures between the groups, and t tests or Mann-Whitney tests to compare continuous measures. To measure baseline differences between the three subgroups, Chi square or Fisher’s exact tests were used to compare categorical measures, and ANOVA with Tukey’s Post-Hoc Tests or Kruskall Wallis tests were used to compare continuous measures. Post-hoc tests for categorical variables were conducted by pairwise comparisons, with a Holm-Bonferroni post-hoc correction.” and “The intervention*time interaction effect was used to measure whether the change over time was different for the web-based CBT compared to the WL. Between-group effect sizes were calculated according to Cohen’s d by subtracting the average difference score between pre-test and post-test of the control group from the corresponding difference score of the web-based CBT group, and dividing the result by the pooled standard deviation of the pre-test. Additionally, the effects over time within the web-based CBT and WL group were measured. Within-group effect sizes were calculated by subtracting the average score at post-test from the average score at pre-test and dividing the result by the pooled standard deviation of the pre-test.”

RESULTS

13a) CONSORT: For each group, the numbers of participants who were randomly assigned, received intended treatment, and were analysed for the primary outcome

Yes. In ‘Participants’ we report that “The remaining 214 participants were randomized to one of the two conditions (web-based CBT or WL), stratified by type of eating disorder (subgroups BN, BED, EDNOS). ….. Within the web-based CBT group, a total of 72 participants (67%) completed the intervention, defined as having completed all 10 assignments and having attended at least 21 sessions; and 36 participants (33%) were considered treatment non-completers. Post-test assignments were completed for 201 participants (94%) with a higher study dropout in the web-based CBT than WL (10% and 2%, respectively; $\chi 2=6.46$, df=1, p=.011).”

13b) CONSORT: For each group, losses and exclusions after randomisation, together with reasons

Yes. A participant flow diagram is included and we also report that “Within the web-based CBT group, a total of 72 participants (67%) completed the intervention, defined as having completed all 10 assignments and having attended at least 21 sessions; and 36 participants (33%) were considered treatment non-completers.”

13b-i) Attrition diagram

Yes. Figure 2 includes information about the percentage of web-based CBT participants of each subgroup that completed the full treatment program (completers) and the percentage of participants who prematurely ended the intervention (non-completers). In ‘Participant flow‘ we also report these numbers including the explanation that participants were considered as completers when they completed all 10 assignments and attended at least 21 sessions. A survival curve or attrition diagram over time has not been included in the current manuscript due to the extensiveness of the information. However, we will give more information about the attrition of participants in another article about the predictors of treatment adherence.

14a) CONSORT: Dates defining the periods of recruitment and follow-up

The date defining the period of recruitment is reported in ‘Participants’: “During the recruitment period of 27 March 2011 to 5 December 2013, 404 subjects were initially interested in participating in the trial.” Furthermore, we have reported that the sampling period was extended, but that the predetermined sample size of 84 participants per eating disorder subtype had not been reached for the subtype BN (n=44). No information is given about the date defining the follow-up period, because not all follow-up data are available yet. We expect that all follow-up data will be available in August 2015. The long-term results of the web-based CBT intervention will be described in another article because the information is too extensive to include in the current manuscript.

14a-i) Indicate if critical “secular events” fell into the study period

This was not applicable to the current study.

14b) CONSORT: Why the trial ended or was stopped (early)

The recruitment period of the trial was stopped early. In ‘Participants’ we report that “As shown in Figure 2, the predetermined sample size of 84 participants per eating disorder subtype had not been reached for the subtype BN (n=44).” and in ‘Limitations’ we mention that “The first and most important limitation is that - despite of extending the sampling period - we were unable to recruit the sample size of 84 participants with BN and therefore also did not reach the planned total sample size of 252 participants.”

15) CONSORT: A table showing baseline demographic and clinical characteristics for each group

Yes; table 1 shows the baseline characteristics of participants.

15-i) Report demographics associated with digital divide issues

Yes; this is shown in table 1.

16a) CONSORT: For each group, number of participants (denominator) included in each analysis and whether the analysis was by original assigned groups

16-i) Report multiple “denominators” and provide definitions

Yes; this is presented in the ‘Results’ section of the manuscript.

16-ii) Primary analysis should be intent-to-treat

Yes; Repeated Measures with Mixed Model analyses were conducted.

17a) CONSORT: For each primary and secondary outcome, results for each group, and the estimated effect size and its precision (such as 95% confidence interval)

Yes; see ‘Results’.

17a-i) Presentation of process outcomes such as metrics of use and intensity of use

17b) CONSORT: For binary outcomes, presentation of both absolute and relative effect sizes is recommended

This was not applicable to the current study.

18) CONSORT: Results of any other analyses performed, including subgroup analyses and adjusted analyses, distinguishing pre-specified from exploratory

Yes; this is presented in the ‘Results’ section / Table 2 of the manuscript.

18-i) Subgroup analysis of comparing only users

This was not applicable to the current study.

19) CONSORT: All important harms or unintended effects in each group

There were no important harms or unintended effects in both groups.

19-i) Include privacy breaches, technical problems

19-ii) Include qualitative feedback from participants or observations from staff/researchers

Participants who prematurely ended the intervention (non-completers) were asked about their reasons for drop-out and for their evaluation of the intervention and therapist. We did not include these results in the current manuscript. However, they will be described in another article because of the extensiveness of this information.

DISCUSSION

20) CONSORT: Trial limitations, addressing sources of potential bias, imprecision, multiplicity of analyses

20-i) Typical limitations in ehealth trials

Yes, in ‘Limitations’ we report several limitations of the current study.

21) CONSORT: Generalisability (external validity, applicability) of the trial findings

21-i) Generalizability to other populations
Yes, in 'Principal Results and comparison With Prior Work' we report that "Because the effectiveness of the web-based CBT was investigated within a naturalistic setting, results of this study are likely to approximate those of the web-based intervention in everyday practice."

21-ii) Discuss if there were elements in the RCT that would be different in a routine application setting

22) CONSORT: Interpretation consistent with results, balancing benefits and harms, and considering other relevant evidence

22-i) Restate study questions and summarize the answers suggested by the data, starting with primary outcomes and process outcomes (use)

Yes, this is reported in the first paragraph of 'Principal Results and Comparison With Prior Work'.

22-ii) Highlight unanswered new questions, suggest future research
Yes, we mention several suggestions for future research based on the results of the current study. These are reported in 'Principal Results and Comparison With Prior Work'.

Other information

23) CONSORT: Registration number and name of trial registry
Yes: Trial Registration: Nederlands Trial Register (NTR): NTR2415; http://www.trialregister.nl/trialreg/admin/rctview.asp?TC=2415 (Archived by WebCite at http://www.webcitation.org/6T2io3DnJ).

24) CONSORT: Where the full trial protocol can be accessed, if available
Yes. In 'Study design and procedure' we report that details of this study have been published previously including a reference to our published study protocol in BMC Psychiatry.

25) CONSORT: Sources of funding and other support (such as supply of drugs), role of funders

Yes. In 'Acknowledgements' we report that "This study received no specific grant from any funding agency, commercial or not-for-profit sectors."

X26-i) Comment on ethics committee approval
Yes. In 'Study design and procedure' we report that "The study protocol was approved by the Ethics Committee of Medical Spectrum Twente in March 2011 (reference number NL31717.044.010, P10-31), and registered on the Dutch Trial Registry (NTR2415; archived by WebCite at http://www.webcitation.org/6T2io3DnJ). Details of this protocol have been published previously [17]."

x26-ii) Outline informed consent procedures

In 'Study procedure and design' we report that written informed consent was obtained: "….they had to provide written informed consent, ….".

X26-iii) Safety and security procedures

X27-i) State the relation of the study team towards the system being evaluated
Yes. In 'Conflicts of Interest' we report that "Elke ter Huurne and Hein de Haan participated in the development of the web-based CBT intervention, but they did not derive financial benefit from the intervention. All other authors declare no conflicts of interest."
