# Supplementary material for: Web-Based Cognitive Behavioral Therapy for Female Patients With Eating Disorders: Randomized Controlled Trial
Source: J Med Internet Res. 2015 Jun 18;17(6):e152. doi: 10.2196/jmir.3946 (PMC4526949; doi:10.2196/jmir.3946)
Supplement: Multimedia Appendix 2 [file jmir_v17i6e152_app2.pdf]

Multimedia Appendix 2. Content of Web-based CBT intervention.

Treatment modules		Content
Part 1		
1	Welcome	Response on the completed baseline questionnaire; additional questions about patient's situation; and an explanation of the intervention.
2	Register daily eating behavior (assignment 1)	Information about the eating diary; and a request to register the eating behavior daily in the eating diary.
3	Description of eating moments (assignment 2)	Response on patient's eating diary; information about eating moments; and a request to describe the moments that the patient feels like eating or has been eating with a detailed description of these situations and patient's thoughts, feelings and behaviors.
4	Analyzing eating situations (assignment 3)	Response on the described eating moments in the eating diary; information about situations that can be a trigger to go eating or feel like eating; and request to complete an assignment about these situations in order to analyze patient's own risk situations.
5	Advantages and disadvantages (assignment 4)	Response on patient's eating diary and the completed assignment; information about the possible advantages and disadvantages of the eating behavior and eating disorder; and a request to report the perceived advantages and disadvantages.
6	Top three	Response on patient's eating diary and the completed assignment ; information about the process of rewarding and the development of habits; and a request to make a list of the three advantages and disadvantages that are most important for the patient.
7	Personal advice	Summary of patient's eating attitudes and behaviors, his/her body image, and the risk situations, thoughts, feelings, and behaviors; personal advice of the therapist; and a request to complete a questionnaire to evaluate patient's opinion about the first part of the intervention and his/her willingness to continue with the second part of the program.
Part 2		
8	Setting goals (assignment 5)	Response on patient's completed evaluation questionnaire and eating diary; feedback about the treatment advise of the multidisciplinary team; and request to complete an assignment about setting goals for eating behavior, exercising patterns, (if applicable) compensatory behavior, and measuring weight.
9	Continuing part 2	Feedback on patient's eating diary and completed assignment about his/her goals for behavioral change and an explanation of part 2 of the intervention.
10	Breaking habits	Response on patient's eating diary; information about the development of habits, short and long term consequences of patient's habits, and possibilities to break these habits.
11	Changing thoughts (assignment 6)	Feedback on patient's eating diary; information about helpful and non-helpful thoughts; and a request to complete the assignment 'Changing thoughts'.
--	Psycho-education or exercise	Response on patient's eating diary and the completed assignment about converting his/her non-helpful thoughts into helpful thoughts; psycho-education or an exercise targeting patient's situation; and a request to judge or discuss this psycho-education or exercise.
12	Changing behavior (assignment 7)	Response on patient's eating diary and his/her feedback on the psycho-education or exercise; information about helpful and non-helpful behavior; and a request to complete an assignment about alternative or helpful behaviors as substitutes for non-helpful behavior.
--	Psycho-education or exercise	Response on patient's eating diary and the completed assignment 'Changing behavior'; psycho-education or an exercise targeting patient's situation; and a request to judge or

		discuss this psycho-education or exercise.
13	Who am I (assignment 8)	Feedback on patient's eating diary and response on psycho-education or exercise; information about self-esteem; and a request to complete an assignment about patient's strengths and weaknesses towards the themes 'work and study', 'daily life', 'mental health' and 'social life' including the instruction to convert these weaknesses into neutral characteristics and related strong and positive characteristics.
--	Psycho-education or exercise	Response on patient's eating diary and the completed assignment 'Who am I'; psycho-education or an exercise targeting patient's situation; and a request to judge or discuss this psycho-education or exercise.
14	Decisions (assignment 9)	Response on patient's eating diary and his/her feedback on the psycho-education or exercise; and a request to complete the assignment 'Decisions' about all decision moments that the patient passes through before relapsing into old eating behavior.
--	Psycho-education or exercise	Response on patient's eating diary and the completed assignment 'Decisions'; psycho-education or an exercise targeting patient's situation; and a request to judge or discuss this psycho-education or exercise.
15	Writing an action plan (assignment 10)	Feedback on patient's eating diary and response on the psycho-education or exercise; information about the thought of stopping, a practical assistant by discontinuing patient's thoughts about eating, compensating behavior or his/her shape; and a request to complete an action plan that will help the patient to maintain his/her behavioral change and prevent that the patient will relapse into old habits.
--	Psycho-education or exercise	Response on patient's eating diary and his/her completed action plan; psycho-education or an exercise targeting patient's situation; and a request to judge or discuss this psycho-education or exercise.
16	Completion of treatment	Summary of patient's progress during the intervention towards his/her goals for eating behavior, exercising patterns, (if applicable) compensatory behavior, and measuring weight; practical information and tips; (if applicable) information about after-care; and a request to complete the post-treatment questionnaire to evaluate the effects and patient's opinion about the intervention.
