# Supplementary material for: Web-Based Cognitive Behavioral Therapy for Female Patients With Eating Disorders: Randomized Controlled Trial
Source: J Med Internet Res. 2015 Jun 18;17(6):e152. doi: 10.2196/jmir.3946 (PMC4526949; doi:10.2196/jmir.3946)
Supplement: Multimedia Appendix 3 [file jmir_v17i6e152_app3.pdf]

Multimedia Appendix 3. Treatment results on secondary outcome measures for participants of the subgroups BN, BED, and EDNOS.

	Web-based CBT (n=108)				WL (n=106)				Interaction effect of group x time[a]			
	Baseline	Post-test	*P*	*d*	Baseline	Post-test	*P*	*d*	F	df	*P*	*d*
BN[b] subgroup (n=44)												
Body dissatisfaction[c]	58.0 (18.6)	51.1 (17.9)	.07	.39	61.9 (16.2)	61.5 (16.7)	.83	.03	2.37	41	.13	.38
Body Mass Index												
<18.5	17.3 (0.2)	17.3 (0.2)	*	*	17.9 (-)	17.9 (-)	*	*	*	*	*	*
18.5 – 25	22.2 (1.8)	23.1 (1.3)	.09	.58	22.0 (1.7)	22.1 (2.8)	.90	.03	1.90	17	.19	.49
>25	29.2 (2.9)	29.4 (2.8)	.56	.07	29.5 (4.0)	30.0 (3.8)	.38	.13	0.03	18	.89	.09
Physical health[d]	25.8 (14.1)	20.2 (12.7)	.005	.43	32.0 (12.3)	30.1 (11.9)	.19	.16	2.83	40	.10	.29
MAP-HSS	12.3 (7.4)	9.8 (6.2)	.005	.37	14.0 (6.1)	13.6 (6.0)	.66	.05	4.03	40	.05	.33
Mental health[e]	40.1 (21.6)	27.6 (20.5)	.006	.60	45.9 (19.9)	42.1 (17.9)	.27	.21	2.71	40	.11	.43
Depression	14.0 (9.1)	9.2 (9.3)	.01	.54	15.7 (8.4)	14.9 (8.4)	.64	.10	2.72	40	.11	.47
Anxiety	7.5 (7.6)	4.9 (5.1)	.07	.41	9.5 (7.2)	8.0 (5.7)	.28	.23	0.36	41	.55	.16
Stress	18.6 (9.0)	13.6 (9.1)	.01	.57	20.7 (9.0)	19.2 (8.5)	.21	.18	2.56	40	.12	.40
Self-esteem[f]	14.1 (7.7)	17.1 (7.6)	.01	.41	14.2 (7.4)	16.8 (5.9)	.005	.39	0.15	40	.70	.07
Quality of life[g]	62.7 (14.8)	69.4 (15.0)	.06	.46	59.2 (19.5)	63.2 (15.3)	.42	.23	0.20	42	.66	.16
Social functioning[h]	8.9 (5.6)	6.6 (4.9)	.02	.45	8.6 (5.5)	8.3 (4.5)	.77	.06	2.11	40	.15	.37
BED[i] subgroup (n=85)												
Body dissatisfaction[c]	60.5 (15.0)	53.9 (19.5)	.006	.38	64.7 (13.0)	64.1 (12.9)	.69	.05	4.78	78	.03	.43
Body Mass Index												
<18.5	*	*	*	*	*	*	*	*	*	*	*	*
18.5 – 25	*	*	*	*	*	*	*	*	*	*	*	*
>25	35.0 (6.5)	34.7 (6.5)	.29	.05	34.2 (5.5)	34.2 (5.4)	.93	.00	0.58	77	.45	.05
Physical health[d]	23.3 (10.7)	17.6 (11.5)	<.001	.53	25.4 (11.9)	24.4 (13.2)	.47	.08	7.88	78	.006	.43
MAP-HSS	12.2 (5.7)	9.6 (6.3)	<.001	.43	13.2 (5.9)	12.6 (6.2)	.30	.13	3.30	78	.07	.31
Mental health[e]	30.4 (16.0)	26.1 (19.1)	.03	.25	36.6 (19.7)	34.6 (21.9)	.54	.10	0.43	78	.52	.13
Depression	10.4 (7.5)	8.7 (7.5)	.16	.23	12.7 (8.2)	12.0 (8.3)	.65	.09	0.27	80	.60	.13
Anxiety	5.4 (5.2)	4.9 (5.9)	.41	.10	7.1 (7.6)	7.9 (7.0)	.47	.11	1.15	80	.29	.21
Stress	14.6 (7.9)	12.3 (8.7)	.02	.28	16.8 (8.6)	14.8 (9.3)	.14	.22	0.05	78	.82	.04
Self-esteem[f]	16.4 (6.6)	18.3 (6.4)	.02	.30	16.4 (6.7)	16.8 (6.5)	.60	.06	2.10	79	.15	.24
Quality of life[g]	61.6 (15.3)	67.9 (16.0)	.02	.41	58.5 (15.9)	62.7 (15.5)	.16	.27	0.31	80	.58	.13
Social functioning[h]	6.7 (4.8)	5.4 (4.1)	.04	.30	6.5 (4.4)	6.5 (4.5)	.89	.01	2.68	80	.11	.28
EDNOS[j] subgroup (n=85)												
Body dissatisfaction[c]	56.5 (12.6)	46.8 (14.9)	<.001	.71	55.0 (14.4)	51.6 (14.2)	.005	.24	6.57	79	.01	.48
Body Mass Index												
<18.5	17.1 (0.4)	17.0 (1.0)	*	*	*	*	*	*	*	*	*	*
18.5 – 25	23.6 (-)	23.6 (-)	*	*	22.1 (2.0)	22.6 (2.0)	.33	.31	0.21	4	.67	.34
>25	33.1 (4.6)	32.7 (4.4)	.22	.06	34.9 (5.2)	34.8 (5.2)	.70	.02	0.43	70	.52	.04
Physical health[d]	20.8 (13.7)	17.7 (13.8)	.003	.23	22.6 (10.2)	21.2 (10.5)	.07	.14	1.91	79	.17	.14
MAP-HSS	10.8 (6.8)	9.1 (6.8)	.01	.25	12.1 (5.8)	11.1 (5.6)	.06	.18	0.78	79	.38	.11
Mental health[e]	28.6 (18.4)	19.2 (16.0)	<.001	.55	26.5 (17.7)	22.1 (17.2)	.005	.25	4.17	80	.04	.28
Depression	10.4 (8.6)	5.5 (5.9)	<.001	.67	8.5 (7.8)	6.1 (7.0)	.001	.33	5.47	80	.02	.32
Anxiety	4.7 (5.2)	4.4 (5.0)	.69	.05	4.3 (5.5)	4.1 (5.5)	.69	.04	0.00	80	1.00	.00
Stress	13.5 (8.6)	9.3 (7.4)	<.001	.53	13.7 (7.5)	12.0 (8.0)	.06	.23	2.93	81	.09	.31
Self-esteem[f]	17.2 (7.3)	19.6 (6.6)	.001	.36	19.7 (7.0)	20.5 (7.3)	.13	.10	3.67	78	.06	.24
Quality of life[g]	64.1 (20.2)	70.5 (16.4)	.01	.35	64.9 (16.5)	69.2 (13.9)	.13	.29	0.34	80	.56	.11
Social functioning[h]	5.9 (4.7)	4.3 (4.1)	.006	.36	5.5 (4.2)	5.1 (3.5)	.45	.09	3.21	80	.08	.28

[a] Treatment outcomes were measured with Repeated Measures and Mixed Model analysis. Effect sizes were measured with Cohen's *d.*
[b] BN = bulimia nervosa.
[c] Body Attitude Test (BAT).
[d] Total score of Maudsley Addiction Profile-Health Symptom Scale (MAP-HSS) and 15 additional eating disorder-specific physical complaints.
[e] Depression Anxiety Stress Scale (DASS).
[f] Rosenberg Self-Esteem Scale (RSES).
[g] EuroQol visual analogue scale (EQ-5D VAS).
[h] Measurements in the Addictions for Triage and Evaluation – International Classification of Functioning, Disability and Health (MATE-ICN).
[i] BED = binge-eating disorder.
[j] EDNOS = eating disorder not otherwise specified.
